# Microwave-Assisted Synthesis of Bioactive Six-Membered Heterocycles and Their Fused Analogues

**DOI:** 10.3390/molecules21040492

**Published:** 2016-04-14

**Authors:** Mohsine Driowya, Aziza Saber, Hamid Marzag, Luc Demange, Rachid Benhida, Khalid Bougrin

**Affiliations:** 1 Laboratoire de Chimie des Plantes et de Synthèse Organique et Bioorganique, URAC23, Faculté des Sciences, Université Mohammed V, B.P. 1014 Rabat, Maroc; m.driowya@hotmail.com (M.D.); aziza.saber@ymail.com (A.S.); marzaghamid@gmail.com (H.M.); 2 Institut de Chimie de Nice, ICN UMR UNS CNRS 7272, Université Nice-Sophia Antipolis-Université Côte d’Azur, Parc Valrose, 06108 Nice Cedex 2, France; 3 Department of Chemistry, Université Paris Descartes, Sorbonne Paris Cité, UFR des Sciences Pharmaceutiques, 4 avenue de l’Observatoire & UFR Biomédicale des Saints Pères, 45 rue des Saints Pères, Paris Fr-75006, France

**Keywords:** microwave irradiation, bioactive molecules, cycloaddition, cyclocondensation, multicomponent reactions

## Abstract

This review describes the formation of six-membered heterocyclic compounds and their fused analogues under microwave activation using modern organic transformations including cyclocondensation, cycloaddition, multicomponents and other modular reactions. The review is divided according to the main heterocycle types in order of increasing complexity, starting with heterocyclic systems containing one, two and three heteroatoms and their fused analogues. Recent microwave applications are reviewed, with special focus on the chemistry of bioactive compounds. Selected examples from the 2006 to 2015 literature are discussed.

## 1. Introduction

**Figure 1:**
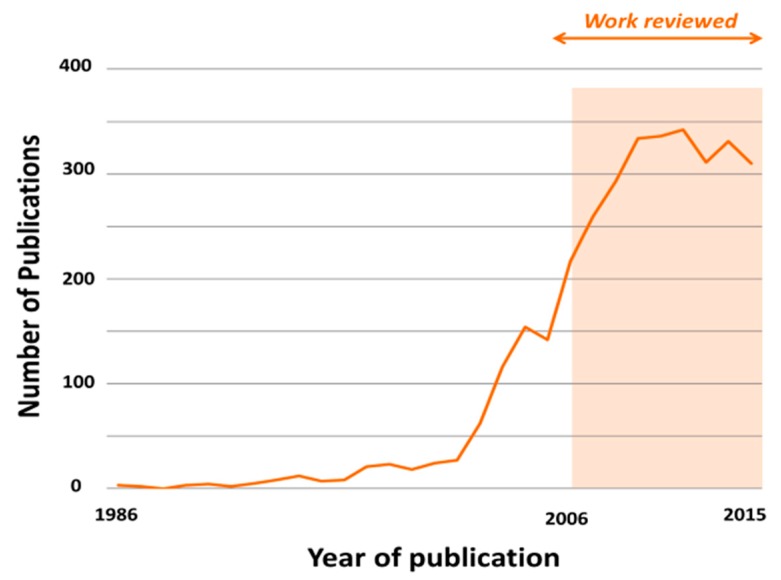
Number of publications dealing with MW-assisted synthesis of heterocycles. This curve has been obtained using “SciFinder^®^”.

Microwave (MW) irradiation is a technique widely used in organic synthesis, as demonstrated by more than 20 years of success stories. Indeed, the growing annual number of publications dealing with this topic is reliable to its importance and popularity (Figure 1). Since the pioneering works of Gedye and Giguere in 1986, more than 6000 articles devoted to MW irradiation have been published [1,2,3,4,5]. The technical accessibility (in terms of costs and simplicity) and the use of very mild conditions to carry out the reactions, associated to the global improvement of the syntheses in terms of conversion rates (yields), shortened reaction times and absence of side-products, contribute today to the huge popularity of the MW irradiation in organic chemistry [6]. In light of this indisputable success, MW has recently come to receive widespread global acceptance in academia and industry.

Heterocycle-containing molecules are extensively studied for their synthesis and their applications not only in medicinal chemistry, but also in optics, electronics and material sciences [7,8]. Therefore, in the last decade, considerable efforts have been performed in designing and carrying out innovative synthetic protocols in heterocyclic chemistry to optimize more eco-sustainable approaches [9].

These processes, also called green chemistry, have been applied not only in the field of medicinal chemistry, but also to natural products and polymer syntheses, material sciences, nanotechnology, essential oil extraction and biochemical processes [10,11,12,13,14]. In addition, the MW-assisted organic syntheses can also lead in short time to large libraries of small-sized heterocyclic compounds featuring high molecular diversity with potential bioactivities [15,16].

MW-assisted syntheses of heterocycles have been extensively reported in the last years, as outlined in Figure 1. Nevertheless, the specific purpose of the present review is to highlight substantive recent applications of MW-irradiation to improve the synthesis of heterocycles formed through cyclocondensation, cycloaddition and other modular reactions. To this end, we selected in this review a panel of new synthetic routes used to prepare highly functionalized heterocyclic compounds with potential bioactivities. We focused this work on the results published in the last decade (2006–2015), which is the most prolific period for this specific topic as illustrated in Figure 1.

In this review, the survey of the 6-membered heterocycles and their fused analogues’ syntheses is arranged according to the number of heteroatoms. Three, four and five-membered ring systems have been previously reviewed by our group and are not addressed in this issue [17]; the synthesis of seven-membered ring and macrocyclic systems will be reviewed by us shortly.

## 2. Synthesis of Heterocyclic Compounds

### 2.1. Six-Membered Heterocycles with One Heteroatom

#### 2.1.1. Pyridines, Dihydropyridines, Piperidines

Pyridines form a class of compounds exhibiting potential activities against a wide range of biological targets [18,19,20]. Its derivatives have been designed and synthesized as therapeutic agents [21,22,23,24], as herbicides, fungicides, pesticides and as fluorescent dyes [25,26]. They have been commonly used as scaffolds for natural product synthesis (e.g., NAD nucleotides, pyridoxol (vitamin B6), and pyridine alkaloids) [27,28,29].

**Figure 2:**
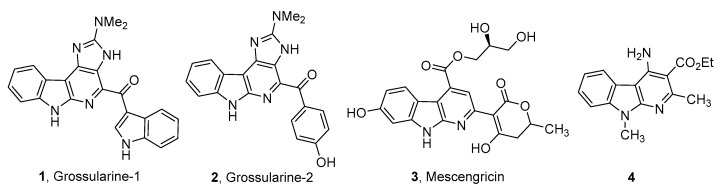
Representative examples of bioactive pyridines.

**Scheme 1:**
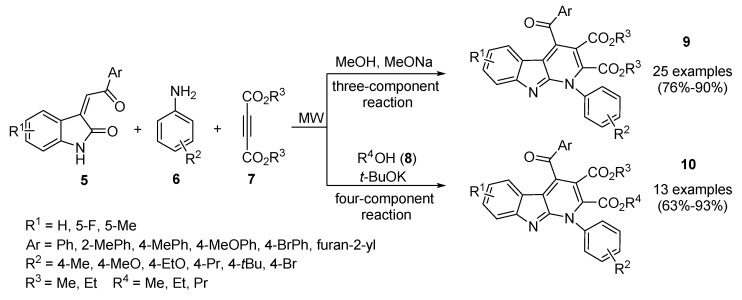
MCR domino reactions leading to polyfunctionalized pyrido[2,3-*b*]indoles.

A wide range of practical syntheses of pyridine derivatives using MW-irradiation have been reported in the last decade [30,31,32,33,34]. In a recent work, Hu *et al.* [30] synthesized a series of novel polyfunctionalized pyrido[2,3-*b*]indoles. This unusual tricyclic scaffold is found in some natural compounds such as grossularine-1 (**1**) and -2 (**2**), mescengricin (**3**) and a GABA modulator **4** having antianxiolitic properties (Figure 2). The reported synthesis by Hu *et al.* involved three- or four-component domino reactions, under MW-irradiation, and in the presence of 3-aroylmethylidene-2-oxindoles **5**, anilines **6**, and acetylenedicarboxylates **7**, with or without alcohols **8**. Interestingly, the authors observed that a selective transesterification only occurs in the case of the four-component reaction (Scheme 1).

**Scheme 2:**
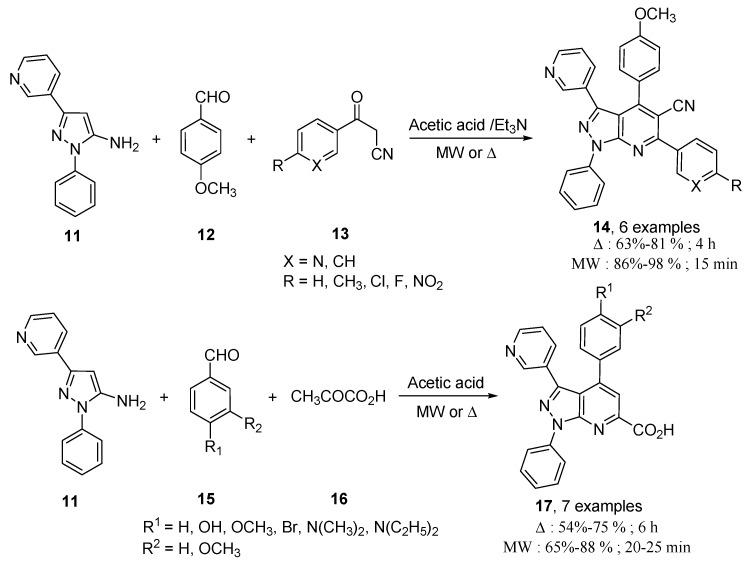
One-pot MCR reaction affording pyrazolo[3,4-*b*]pyridines.

On the other hand, El-Borai *et al.* [31] described a new synthesis of pyrazolo[3,4-*b*]pyridine derivatives through a one-pot multi-component reaction. In this synthesis, 5-amino-1-phenyl-3-(pyridin-3-yl)-*1H*-pyrazole (**11**) was allowed to react with either 4-anisaldehyde (**12**) and *p*-substituted α-keto-nitriles **13** or with pyruvic acid (**16**) and some aromatic aldehydes **15** in acetic acid (Scheme 2). The reaction was carried out by conventional heating and under MW irradiation. The authors reported that the use of MW lead to shortened reaction times and higher yields compared to those obtained by conventional heating.

These newly synthesized compounds were screened for their antibacterial activity against Gram-positive bacteria (*Bacillus*) and Gram-negative bacteria (*Escherichia coli*, *Enterobacter cloaca* and *E. serratia*), and also for their antifungal activity against *Fusarium oxysporum* and *Penicillium expansum*. Most of the compounds showed higher antibacterial potencies against the Gram-negative bacteria rather than the Gram-positive bacteria and fungi.

**Scheme 3:**
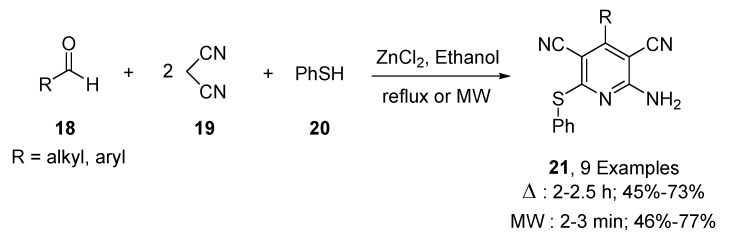
First example of acid-catalyzed synthesis of 6-Amino-3,5-dicarbonitrile-2-thio-pyridines.

6-Amino-3,5-dicarbonitrile-2-thio-pyridines **21** are usually synthesized under base catalysis. The first example of such a reaction catalyzed by a Lewis acid was recently reported by Sridhar *et al.* [32]. This synthesis was carried out by means of an efficient one-pot multi-component reaction, which combines aliphatic, aryl, or heteroaryl aldehydes **18** with malononitrile (**19**) and thiophenol (**20**) in the presence of ZnCl_2_ either under MW-irradiation or conventional heating conditions. In both cases, the compounds were obtained in moderate to good yield; nevertheless, MW-irradiation allowed shortened reaction times (Scheme 3).

**Scheme 4:**
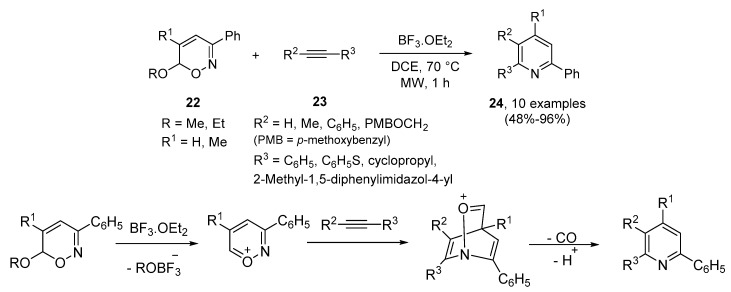
Synthesis of functionalized pyridines through [4 + 2] cycloaddition.

Another new method allowing the preparation of highly functionalized pyridine derivatives was established by Linder *et al.* [33]. In this synthesis, *6H*-1,2-oxazines **22** and various alkynes **23** reacted in the presence of a Lewis acid catalyst and under MW-irradiation leading to polyfunctionnalized pyridines **24** (Scheme 4).

This procedure leads to higher yields compared to conventional syntheses. The authors hypothesized that the addition of the Lewis acid (TiCl_4_ or BF_3_·OEt_2_) on the oxazine induced the formation of an 1,2-azapyrylium ion which remains stable even at high temperature. Moreover, the authors postulated that MW-irradiation is mandatory for completing its formation. Next, this azapyrylium ion reacts with substituted alkyne in a [4 + 2] cycloaddition to give a bridged intermediate. The last step of the reaction is a retro Diels-Alder reaction, leading to the expected pyridines. 

**Scheme 5:**
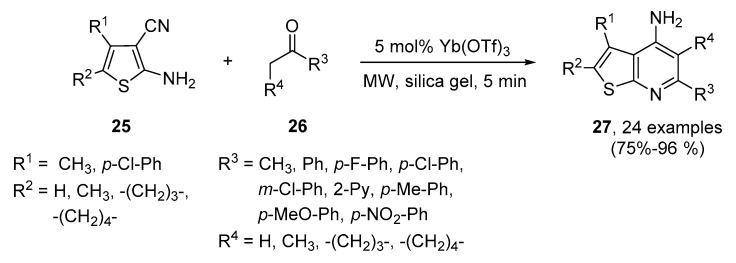
Solvent-free synthesis of functionalyzed thieno[2,3-*b*]pyridines.

A novel one-step synthesis of thieno[2,3-*b*]pyridine derivatives **27** catalyzed by ytterbium(III) triflate under solvent-free conditions was reported by Wieke *et al.* [34]. Thus, in the presence of a catalytic amount of Yb(OTf)_3_ and under MW irradiation with silica gel, several series of 2-amino-3-thiophene-carbonitriles **25** reacted easily with different ketones **26** to afford, in only 5 minutes, the corresponding amino-thieno[2,3-*b*]pyridines **27** in good to excellent yields. Importantly, the catalyst could be easily recovered and reused several times (Scheme 5).

1,4-Dihydropyridine derivatives have a wide range of biological and pharmaceutical activities and are also involved in hydride transfer from the reduced nicotinamide adenine dinucleotide (NADH and NADPH) coenzymes, and analogues thereof, which mediate hydrogen transfer reactions in biological systems [35,36,37].

**Scheme 6:**
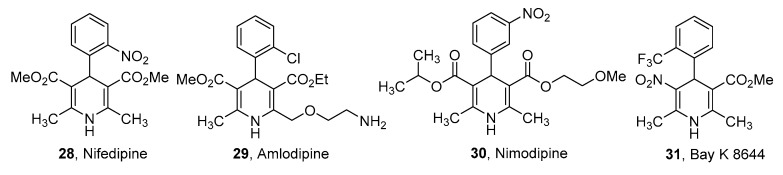
Representative examples of bioactive 1,4-dihydropyridines

Among the 1,4-dihydropyridine derivatives, nifedipine (**28**), amlodipine (**29**) and nimodipine (**30**) [38,39] (Scheme 6) are potent calcium channel antagonists, used clinically for the treatment of cardiovascular diseases such as hypertension and angina pectoris [40]. In addition, others 1,4-dihydropyridines have been discovered as potent calcium channel agonists (such as BAY K 8644 (**31**)) [41,42].

**Scheme 7:**
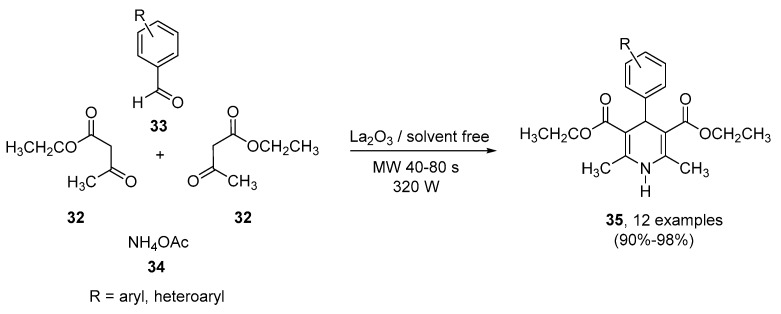
Solvent-free three-component reaction leading to 1,4-dihydropyridines.

Surprisingly, very few examples of 1,4-dihydropyridines syntheses under MW-irradiation have been described. Kuraitheerthakumaran *et al.* [43] reported a simple and efficient three-component protocol starting from β-ketoester **32**, aldehydes **33** and ammonium acetate (**34**). The reactions were performed under solvent-free conditions using lanthanum oxide as a catalyst. This new method provided the expected products **35** in excellent yields (90%–98%) after short reaction times (40–80 s) in comparison to the classical Hantzsch methods (Scheme 7) [44,45,46].

**Scheme 8:**
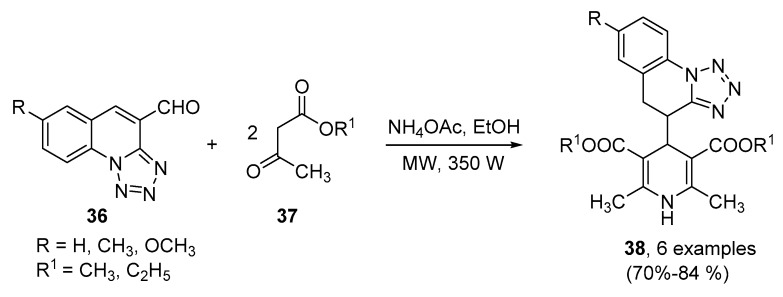
MW-assited Hantzsch reaction affording 1,4-dihydropyridines.

Ladani *et al.* [47] synthesized new 1,4-dihydropyridine derivatives by a MW-assisted Hantzsch condensation *via* three-components reactions involving tetrazolo[1,5-*a*]quinoline-4-carbaldehydes **36**, ethyl/methyl acetoacetate **37** and ammonium acetate (Scheme 8). The resulting series of 1,4-dihydropyridines **38** were screened for their antimicrobial activity. Some of these newly synthesized compounds showed better fungicidal activity, specifically against *R. oryzae*, than the standard drugs ampicillin and griseofulvin. Conversely, they displayed poor bactericidal activity.

**Figure 3:**
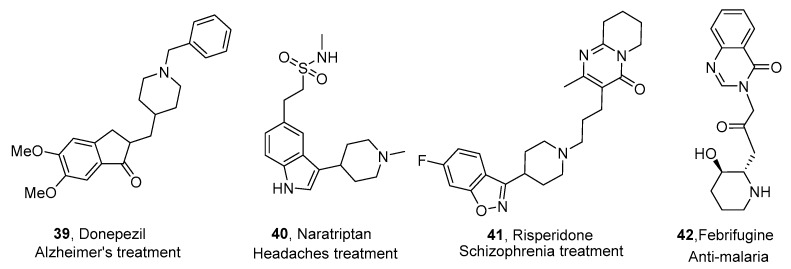
Representative examples of bioactive piperidines.

Compounds containing a piperidine moiety represent a large class of natural products and their syntheses have become an interesting topic due to their relevance as bioactive components in pharmaceutical science in recent years (Figure 3) [48,49,50].

**Scheme 9:**
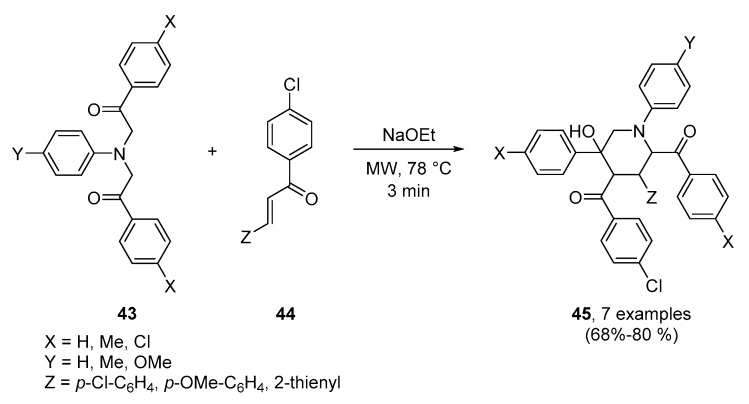
Synthesis of pyridines through one-pot procedure.

An interesting example of piperidine synthesis has been described by Ravindran *et al.* [51]. This approach proceeded through the condensation of diphenacyl anilines **43** with different arylidene acetophenones **44** in the presence of a catalytic amount of sodium ethoxide. This one-pot tandem sequence involved a Michael addition-aldol reaction ring closure and afforded highly substituted piperidines **45** in good yields (Scheme 9).

#### 2.1.2. Quinolines

**Figure 4:**
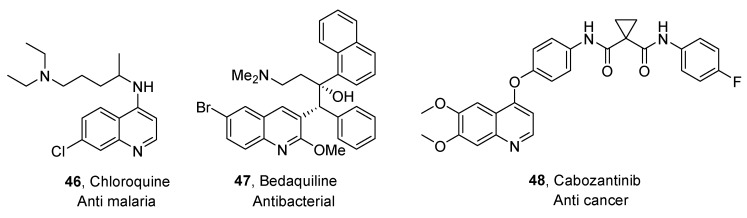
Representative examples of bioactive quinolines.

Quinolines and their derivatives are important heterocyclic compounds because of their wide-ranging biological activities [52,53,54] and interesting photochemical properties [55]. For example, chloroquine (**46**) has been used for its antimalarial activity for more than 60 years; [56,57,58] bedaquiline (**47**), an inhibitor of the mycobacterial ATP synthase, has been approved to treat multi-drug resistant tuberculosis, [59] and cabozantinib (**48**), a multitargeted receptor tyrosine kinase inhibitor, showed effective anticancer activity and has been marketed for the treatment of medullary thyroid cancer (Figure 4) [60].

**Scheme 10:**
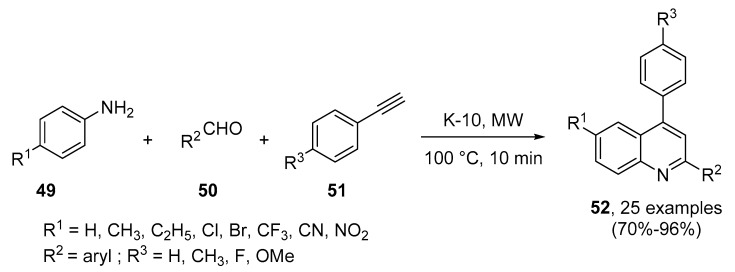
Solid acid-catalyzed synthesis of substituted quinolines.

Therefore, in the last decade, several new synthetic routes to quinoline derivatives under MW-irradiation have been reported [61,62,63,64,65,66]. Kulkarni *et al.* [61] described a solid acid-catalyzed synthesis of substituted quinolines via a MW-assisted three-component domino reaction between anilines, aldehydes and terminal aryl alkynes. The reaction was catalyzed by montmorillonite K-10, a strong and environmentally safe solid acid and performed under solvent-free conditions. The synthetic pathway involved the formation of an imine by condensation of *para*-substituted anilines **49** and aldehydes **50**, followed by nucleophilic attack of phenylacetylene **51** on the formed imine, intramolecular cyclization and aromatization. The K-10 catalyst was recovered during five successive reaction cycles, remained very stable, showed no sign of deactivation and provided excellent yields. The combination of solid acid catalysis, multicomponent domino reaction approach and microwave irradiation provided the quinolones **52** in excellent selectivities and short reaction times (Scheme 10).

**Scheme 11:**
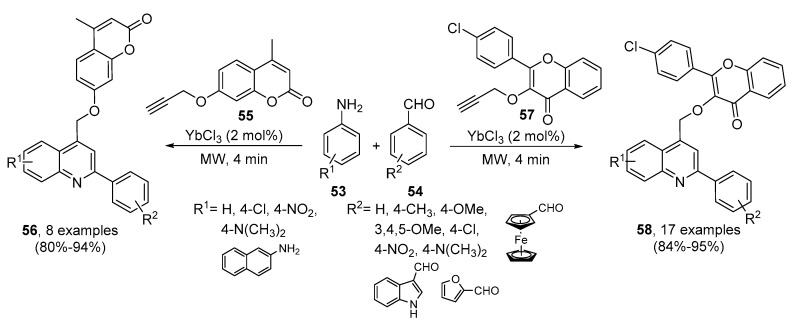
One-pot green synthesis of quinolin-4-ylmethoxy-chromen-2/-4-ones.

Another green-chemistry related approach was recently reported by Kumar *et al.* [62] for the synthesis of novel quinolin-4-ylmethoxy-chromen-2/-4-ones **56**, **58** under MW conditions. The protocol involves a one-pot reaction of aromatic amines **53** and aldehydes **54**, and a propargylated-flavone (**55**) or -coumarin (**57**) using YbCl_3_ (2 mol%) as catalyst. The reactions were carried out at 100 °C, and led to the expected products with excellent yields after a short reaction time (4 min). The authors hypothesized a three step mechanism: domino-imine formation between the aldehyde and the amine, activation of the imine by the catalyst and subsequent addition to the acetylenyl derivative leading to a cyclization, and finally oxidation of the intermediate. According to this model, Yb^3+^ as catalyst enhances the electrophilicity of the imine, and therefore plays a key role either in imine formation and cyclization steps. Importantly, the reaction occurs in water, which does not deactivate nor decompose the catalyst (Scheme 11).

These highly functionalized quinolone derivatives have been examined for their *in vitro* antibacterial activity against Gram-positive (*S. aureus* and *B. subtilis*) and Gram-negative (*E. coli* and *S. flexneri*) bacteria, and also for their antifungal potential against *Candida albicans*. Their activities were compared to those of marketed drugs, *i.e.*, ampicillin and cefadroxil (antiobiotic) and fluconazole (antifungal). Several compounds exhibited better or equally potent antibacterial potencies, expressed as *in vitro* minimum inhibitory concentration values (MIC), as the reference drugs against the four bacteria of the panel (MIC values in the 0.4–6 mg/mL range). The structure activity relationship study highlights the relevance of the heterocyclic moiety of the quinoline scaffold. Promising results have been also obtained in anti-fungal assay, since one compound exhibited a MIC value of 0.4 mg/mL, which is ten times better than fluconazole.

**Scheme 12:**
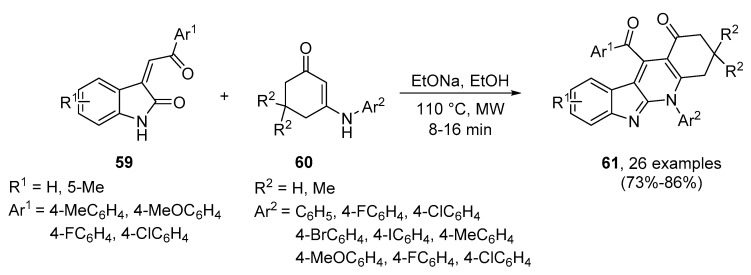
Domino reactions leading to indolo[2,3-*b*]quinolones.

Very recently, Li *et al.* [63] have developed a new synthesis of tetracyclic indolo[2,3-*b*]quinolone derivatives **61** via domino heterocyclization of 3-aroylidene-2-oxindoles **59** with enaminones **60** in a sealed vessel under microwave irradiation. The reaction cascades consisted of an initial Michael addition, tautomerism, intramolecular cyclization, and dehydration leading to aromatization (Scheme 12). This methodology showed attractive properties, such as short reaction times, high yields and operational simplicity.

**Scheme 13:**
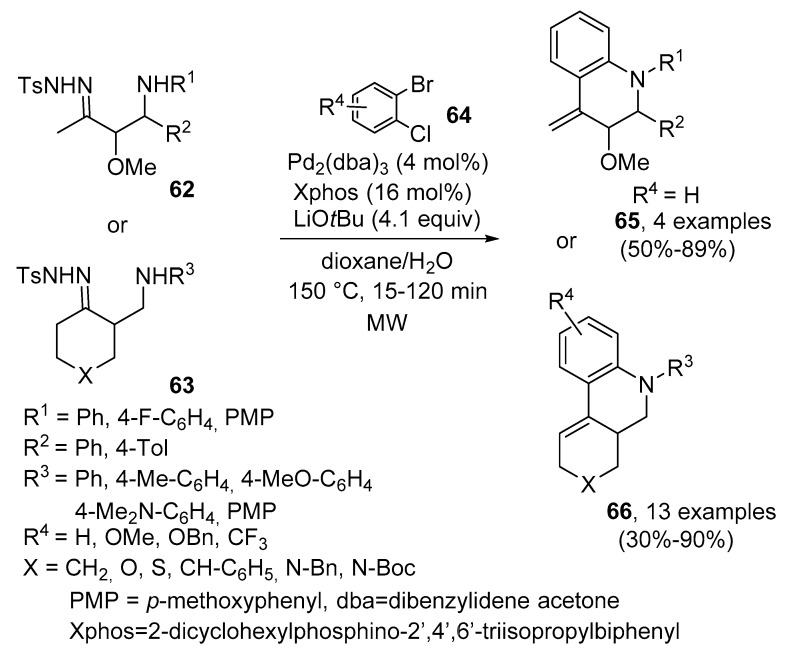
Tetrehydroquinolines synthesis through palladium catalyzed cross-coupling/intramolecular C-N bond forming tandem reaction.

Barluenga *et al.* [64] have developed the synthesis of tetrahydroquinoline derivatives **65**, **66** by tandem palladium catalysis under MW irradiation. This method is based on the cross-coupling of tosylhydrazone derivatives **62**, **63** with 1,2-dihalogenated aromatic benzenes **64** under Pd catalysis, followed by an intramolecular C-N bond-forming reaction (Scheme 13). The reaction took between 15 and 120 min, and provided a series of isoquinoline derivatives in low to good yields (30%–90%).

**Scheme 14:**
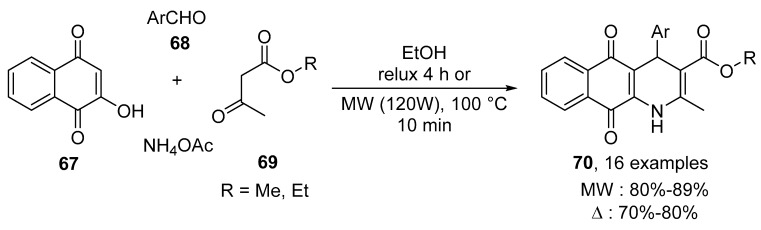
Hantzsch condensation leading to tetrahydrobenzo[*g*]quinolones.

**Scheme 15:**
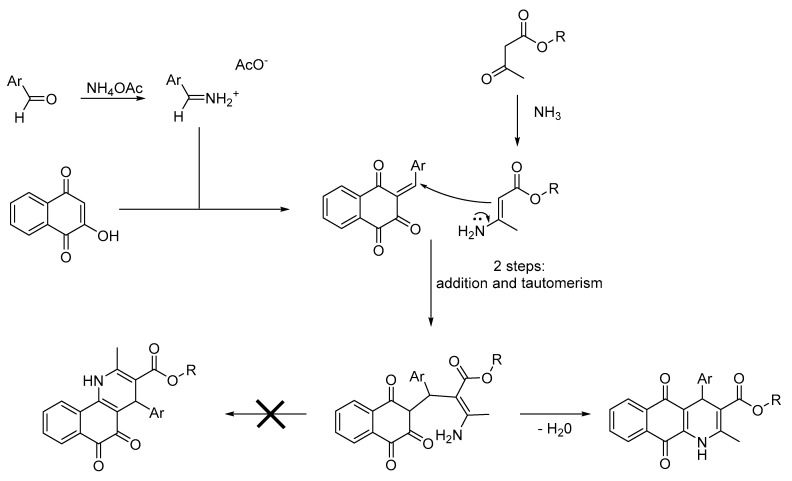
Mechanistic study of the tetrahydrobenzo[*g*]quinolone synthesis reported by Devi Bala *et al.* [65].

Devi Bala *et al.* [65] have described a new Hantzsch condensation method for the regioselective synthesis of a library of tetrahydrobenzo[*g*]quinolines under microwave irradiation (Scheme 14). The products were obtained in excellent yields and short reaction times, starting from 2-hydroxy-1,4-naphthaquinone (**67**), aromatic aldehydes **68**, methyl or ethyl acetoacetate **69** and ammonium acetate in ethanol as solvent. All reactions were performed in a sealed vial and irradiated in a focused microwave at 100 °C, or conducted by classical heating in an oil bath for 4 h. The mechanistic study of this reaction points out the total regioselectivity of the intramolecular condensation, which occurs during the last step. This is probably due to the higher electrophilicity of the carbonyl at the non-conjugated 3-position related to these of the carbonyl at the conjugated 1-position (Scheme 15).

This synthetic pathway showed a significant acceleration of the reaction under MW (10 min) compared to the conventional heating (4 h), and leads to the expected tetrahydrobenzo[*g*]quinolones **70** in high yields.

**Scheme 16:**
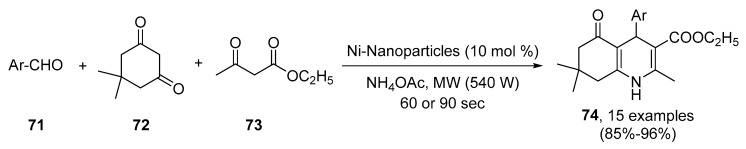
Synthesis of polyhydro-quinolines through a solvent-free Hantzsch procedure.

The use of nickel nanoparticles as a heterogeneous catalyst for the synthesis of polyhydro-quinoline derivatives **74** via solvent-free Hantzsch condensation was described by Sapkal *et al.* [66]. In this work, a one-pot four component reaction involving aromatic aldehydes **71**, dimedone (**72**), ethyl acetoacetate (**73**) and ammonium acetate led to polyhydroquinoline derivatives in excellent yields after short reaction times (Scheme 16). All reactions were performed using nanosized nickel particles and microwave activation. Moreover, the catalyst was recovered and reused for the same reaction during four consecutive runs without any apparent loss of activity.

#### 2.1.3. Pyrans

**Scheme 17:**
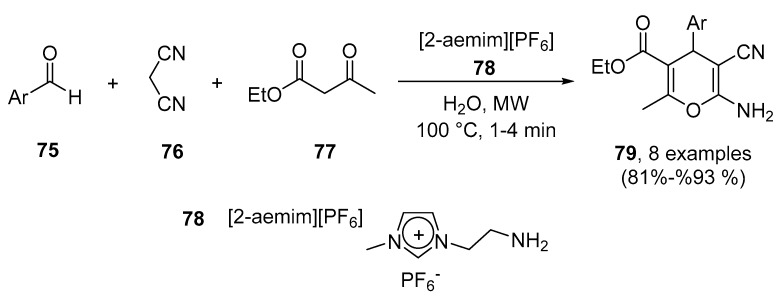
One-pot synthesis of 4*H*-pyrans using a mixture of water and ionic liquid as solvent.

A huge variety of bioactive compounds include a pyran ring, especially sugars, and several pyrene derivatives. They feature a broad-spectrum bioactivities such as anti-inflammatory, sedative, anti-tubercular, anti-protozoal, anti-bacterial and anti-fungal properties [67,68,69,70]. New strategies for the synthesis of pyrene derivatives have been developed in recent years [71,72]. Peng and Song [72] have used the ionic liquid [2-aemim][PF_6_] (1-methyl-3-(2-aminoethyl)imidazolium hexafluorophosphate) for the one-pot synthesis of 4*H*-pyran derivatives under focused MW-irradiation. A mixture of aromatic aldehydes **75** with malononitrile (**76**) and ethyl acetoacetate (**77**) was reacted at 100 °C using a combination of [2-aemim][PF_6_] (**78**) and water as solvent, and the microwave-mediated cyclization occurred rapidly (1–4 min). The expected 4*H*-pyran derivatives **79** have been isolated in excellent yields (Scheme 17). Moreover, the aqueous amino-functionalized ionic liquid has been recycled, and the same batch of solvent has been used more than seven times.

**Figure 5:**
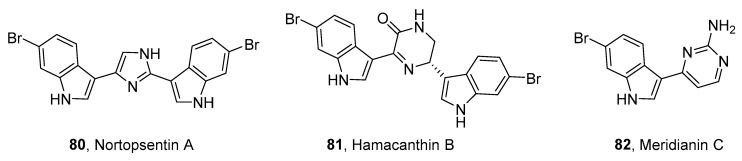
Representative examples of bioactive indolyl-pyran natural prodcuts.

A very interesting chemioselective synthesis under MW-activation of highly functionalized indolyl-pyran derivatives has been reported by Kamalraja *et al.* [72]. These synthetic pyrans are described as analogues of some important bioactive compounds such as nortopsentins A–C (**80**); [73,74] hamacanthin B (**81**); [75,76] meridianins A–E (**82**) (Figure 5) [77,78].

**Scheme 18:**
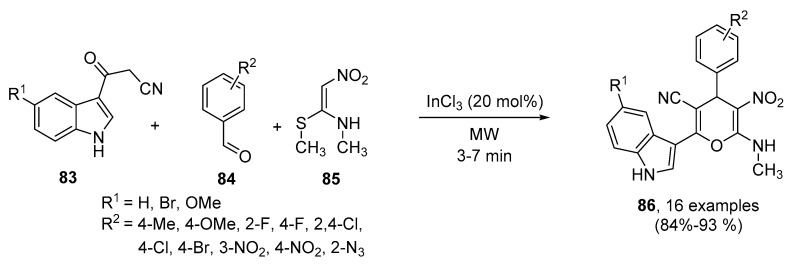
Three-component solvent-free coupling reaction leading to indolyl-pyrans.

**Scheme 19:**
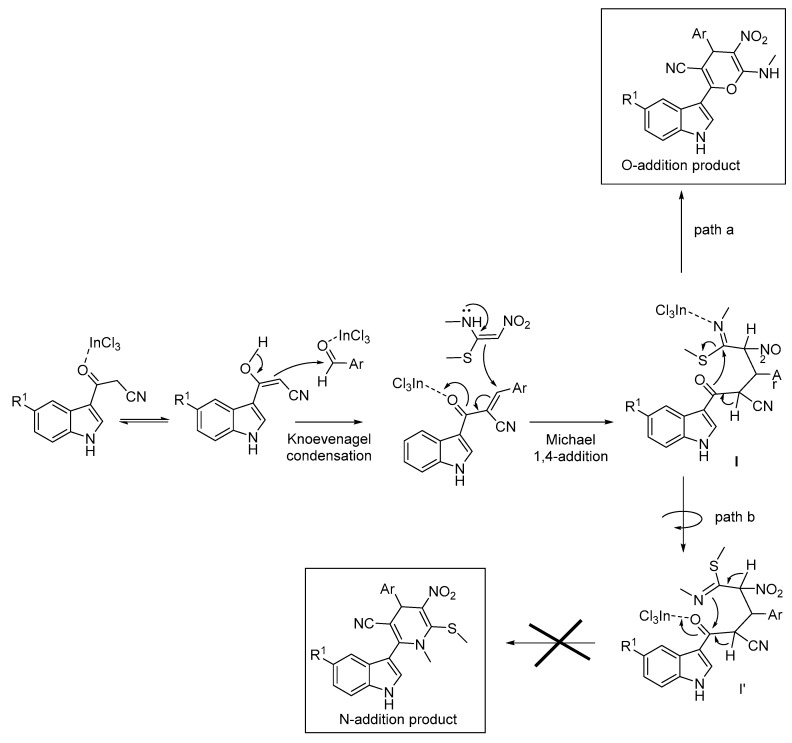
Mechanistic study of the indolyl-pyrans synthesis reported by Kamalraja *et al.* [72].

The procedure consists of a three-component solvent-free coupling of substituted 3-cyanoacetyl indoles **83**, various aromatic aldehydes **84**, and (*E*)-*N*-methyl-1-(methylthio)-2-nitroethenamine (**85**), in the presence of InCl_3_ as a catalyst. The syntheses were completed within 3–7 min and the pure indolylpyrans **86** were isolated in high yields (Scheme 18). According to the authors’ hypothesis, this reaction is divided in three steps (Scheme 19). Firstly, the 3-cyanoacetylindole is tautomerized, and condensed with the aromatic aldehyde through a Knoevenagel condensation. The resulting intermediate, a Michael acceptor, which undergoes the addition of (*E*)-*N*-methyl-1-(methylthio)-2-nitroethamine, affording the open-chain intermediate **I**. Lastly, this intermediate is cyclized through a *O*-addition according to path a. However, intermediate **I** is also in equilibrium with an alternative rotameric form (**I**’), whose *N*-cyclization, according to path b, could lead to dihydropyridine. The authors reported that they never observed the formation of this latter adduct, underlying the exceptional regio- and chemoselectivities of their original synthetic route.

#### 2.1.4. Benzopyrones: Coumarins and Chromones

Benzopyrones are an important class of oxygenated heterocycles, widely distributed in plants, including edible vegetables and fruits [79] and many of them have broad and interesting ranges of biological activities [80,81,82,83]. The two more important benzopyrone derivatives are the chromones and coumarins.

**Figure 6:**
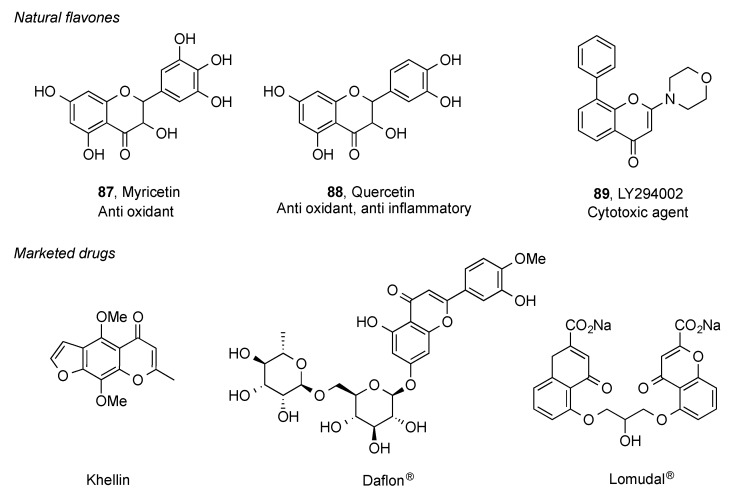
Representative examples of bioactive chromones.

Chromone (chromen-4-one or 1,4-benzopyrone) derivatives, exemplified by compounds like **87**–**89** (Figure 6) are widespread in Nature, particularly in flowers as pigments, and have interesting biological activities such as antiviral, [84], antioxidant [85,86], anti-inflammatory [87,88], and anticancer properties [89]. Some of them have been also reported as kinase inhibitors [90,91]. Several chromone derivatives have been reported for their clinical use as therapeutic agents. For example, khellin (Figure 6), a natural product extracted from the seeds of the plant *Ammi visnaga*, was the first chromone-based therapy mainly used for centuries in the Mediterranean medicine as a diuretic, relaxant and also in the treatment of angina, asthma and vitiligo, a melanoma-driven pigmentation [92,93,94]. Daflon^®^ (diosmin) is a venotropic drug used in the treatment of venous diseases and hemorrhoids, while Lomudal^®^ (sodium cromoglycate) is clinically used for the treatment of asthma and allergies (Figure 6) [95,96].

**Scheme 20:**
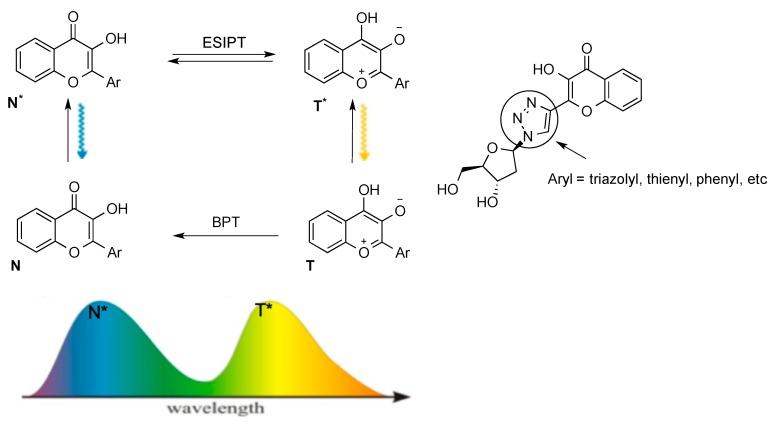
Use of hydroxychromones as ratiometric dyes.

Besides their pharmacological interest, several chromones, particularly 3-hydroxychromones, also exhibit interesting fluorescence properties. They have been used for several applications such as protein and nucleic acid labelling [97,98]. Recently, 2-aryl-3-hydroxychromones have been also studied as ratiometric dyes [99,100]. Indeed, 2-aryl-3-hydroxychromones when excited give rise to two emission bands induced by an Excited State Intramolecular Proton Transfer (ESIPT) process, Scheme 20). Upon excitation, the normal excited molecules (N *) may undergo the ESIPT reaction producing the tautomer (T *) excited form. Both states are highly emissive, the T* band being in general red-shifted comparing to the N * band, as reported for triazolyl- and thienyl-hydroxychromones (Scheme 20) [100,101,102].

**Figure 7:**
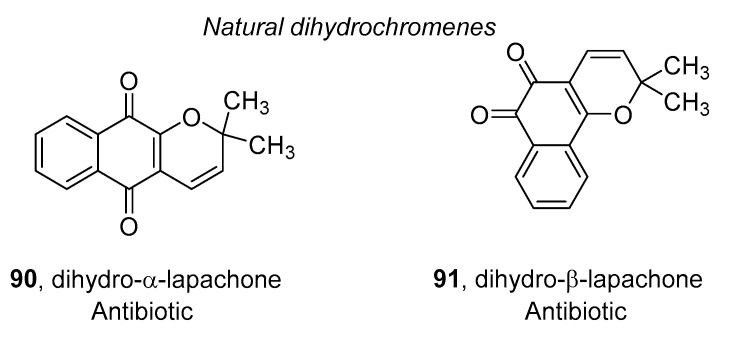
Representative examples of bioactive dihydrochromenes.

3,4-Dihydro-α-lapachone (**90**), and its isomer 3,4-dihydro-β-lapachone (**91**) are the most important members of the dihydrochromene derivatives coupled with *ortho*- and *para*-quinones. These natural products have been isolated from many varieties of plants, fungi, and insects [103]. Their antibiotic activity has been successfully used against the Gram-negative bacteria genus *Brucella* [104], and against multidrug-resistant strains of *Staphylococcus aureus* [105]. Dihydro-α-lapachone inhibits the mycelial growth of several fungi (such as *B. cinerea*, *Colletotrichum acutatum* Simmonds and *M. grisea*) and is active *in vivo* on rice and tomato plants [106,107] (Figure 7).

**Figure 8:**
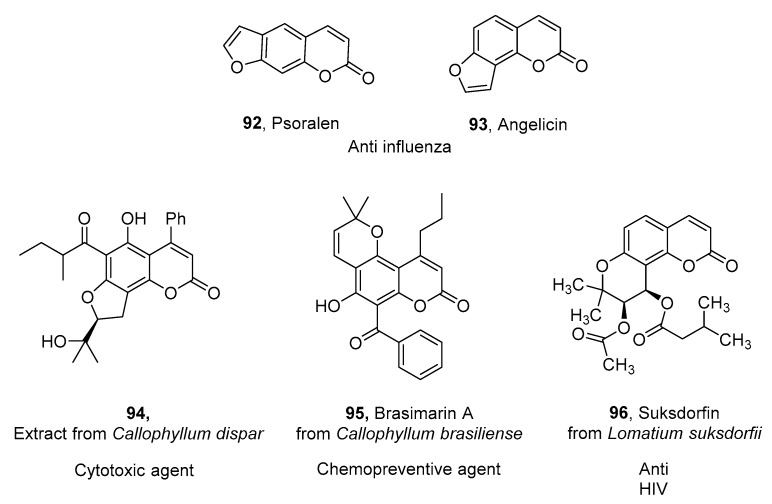
Representative examples of bioactive coumarines.

The coumarin (1,2-benzopyrone) scaffold occurs in a wide variety of plant extracts including cassia, lavender, yellow sweet clover and woodruff [108]. It also found in fruits (e.g., bilberry, cloudberry), green tea and other foods such as chicory. Coumarin derivatives possess a remarkable range of biological properties including antioxidant [109,110], vasorelaxant [111], antiviral (**92**, **93**) [112,113], anti-cancer (**94**, **95**), and anti-inflammatory activities [114,115]. Lastly, suksdorfin (**96**), another natural compound isolated from the fruit of *Lomatium suksdorfii*, exhibits a significant anti-HIV activity as it inhibits the viral replication in the T-cell line with an EC_50_ in the 2.5 µM range [116] (Figure 8).

**Scheme 21:**
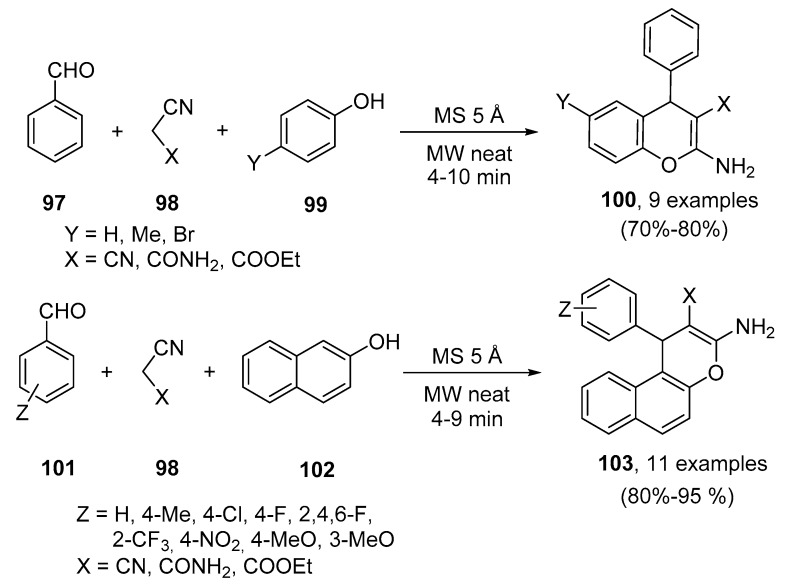
Chromenes and benzochromenes synthesis through one-prot three-component reactions.

Different synthetic methods have been developed for the preparation of the chromene derivatives. Shekhar *et al.* [117] have reported an efficient green synthesis of substituted *4H*-1-benzopyrans (chromenes) and 1*H*-naphtho[2,1-*b*]pyrans (benzochromenes) via a one-pot three-component condensation of a phenol **99** or naphthalen-2-ol (**102**), an aromatic aldehyde **97**, **101**, and an active methylene-containing compound **98**. The reaction was carried out in the presence of 5 Å molecular sieves as an inexpensive catalyst under solvent-free microwave irradiation conditions (Scheme 21). The formation of the benzopyran products involved an initial Knoevenagel reaction between the aldehyde and the activated methylene derivative to afford the arylidene intermediate. This step is followed by the alkylation of naphthalen-2-ol or phenol derivatives and the final cyclization. A library of new benzopyrans products **100**, **103** was obtained in excellent yields (70%–95%) after short reaction times (4–15 min).

**Scheme 22:**
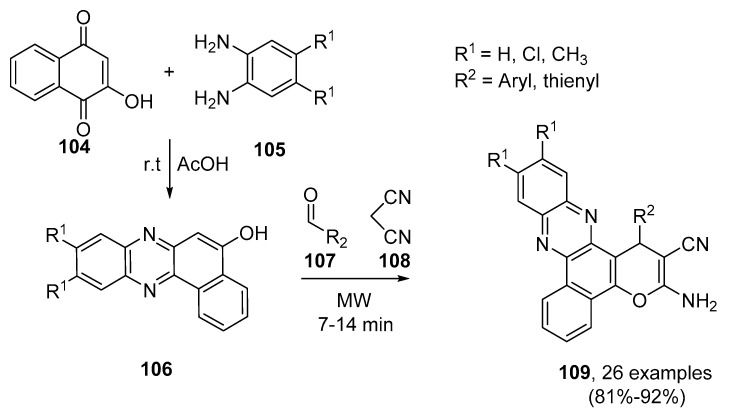
One-pot two-step tandem synthesis of benzo[*a*]pyrano[2,3-*c*]phenazines.

In a similar approach, Wang *et al.* [118] have reported a new one-pot two-step tandem synthesis of highly functionalized benzo[*a*]pyrano[2,3-*c*]phenazine derivatives **109** under MW-irradiation. The authors used this method for the combinatorial synthesis of a benzo[*a*]-pyrano[2,3-*c*]phenazine libraries, which are analogues of biologically active compounds [119,120,121]. This reaction was achieved by the simple condensation of 2-hydroxynaphthalene-1,4-dione (**104**), diamines **105**, aldehydes **107** and malonitrile (**108**). Expected compounds are obtained in short reaction times and in good to excellent yields (Scheme 22).

**Scheme 23:**
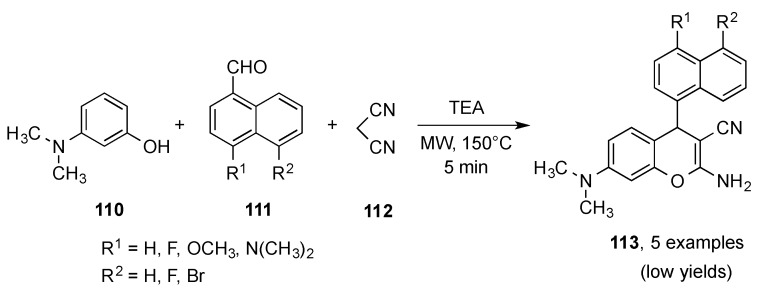
One-step procedure leading to chromenes.

Patil *et al.* [122] have described a fast and efficient method to synthesize substituted chromenes **113** using MW-activation. The reaction was achieved in a single step by the cyclocondensation of 3-dimethylaminophenol (**110**), substituted naphthaldehydes **111** and malononitrile (**112**) with few drops of TEA and MW-irradiated at 150 °C for 5 min under pressure. In a preliminar assay, several chromenes showed high anti-proliferative activities (IC_50_ in the 7–640 nM range) against several human cancer cell lines (including melanoma, prostate and glioma). Moreover, these molecules do not exert cytotoxicity against normal astrocytes (Scheme 23).

**Scheme 24:**
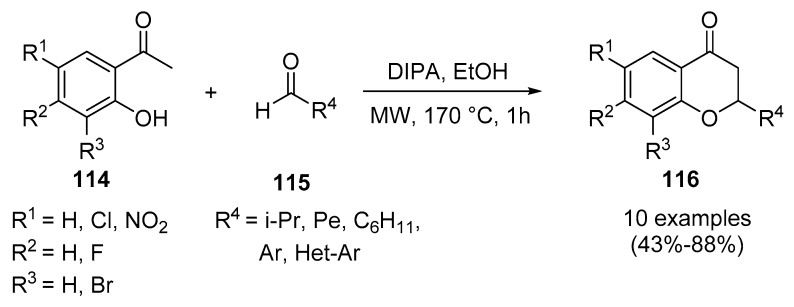
Base-promoted synthesis of 2-alkyl-substituted 4-chromanones.

To synthesize a diverse set of 2-alkyl-substituted 4-chromanones **116**, Fridén-Saxin *et al.* [123] have developed an efficient method, which consisted on a base-promoted condensation of 2-hydroxyacetophenones **114** and various aldehydes **115**. These reactions were performed using diisopropylamine in ethanol at 170 °C and afforded the required products in moderate to high yields within one hour (Scheme 24).

These substituted chromones have been evaluated as novel inhibitors of the NAD-dependent deacetylase sirtuin-2 (SIRT2), which deregulation is involved in age-related diseases (e.g., neurodegenerative disorders). In this study, 6,8-dibromo-2-pentyl-chroman-4-one has been highlighted as the best SIRT2 inhibitor, exhibiting an IC_50_ of 1.5 μM [124].

**Scheme 25:**
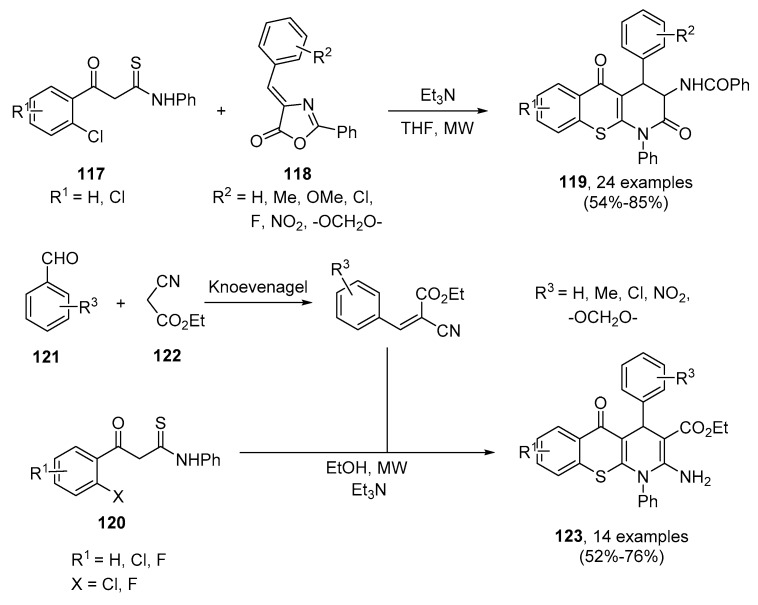
Domino reactions leading to thiochromones.

**Scheme 26:**
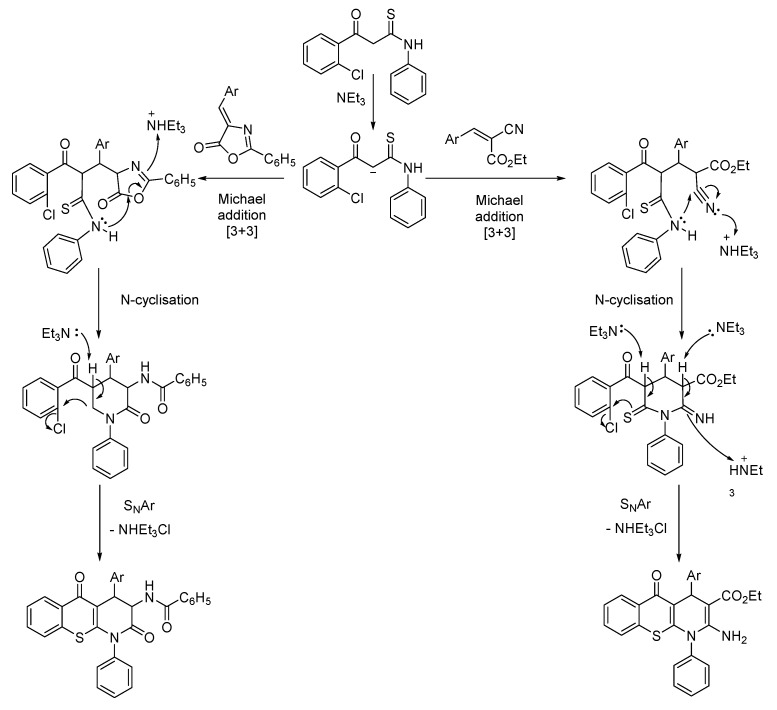
Mechanistic study of the thiochromones synthesis reported by Wen *et al.* [125].

The synthesis of thiochromone derivatives has been studied by Wen *et al.* [125], who reported two new strategies to synthesize series of unusual fused tricyclic thiochromeno [2,3-*b*]pyridines **119** and **123** in good yields. These derivatives were obtained by domino reaction involving β-(2-chloroaroyl) thioacetanilides **117**, **120**, activated 4-arylidene-2-phenyloxazol-5(4*H*)-ones **118** or aromatic aldehydes **121** and ethyl 2-cyanoacetate (**122**) under MW-irradiation (Scheme 25). The authors hypothesized that this domino process occurs in three successive steps (Scheme 26). The first reaction consists in a Michael addition of the thioacetanilide anion to the oxazolone (or to the cyanophenylacrylate intermediate obtained through the Knovenagel condensation of various aromatic aldehydes with ethyl 2-cyanoacetate). The resulting intermediate undergoes an *N*-cyclization, and lastly the *o*-chlorine of the aryl group is substituted by the mercapto group through an intramolecular nucleophilic substitution (S_N_Ar). Importantly, no side products other than HCl, which is trapped by the trimethylamine, are formed through this tandem [3+3] annulation procedure and the final compounds are easily purified by recrystallisation. In addition, the authors have demonstrated the efficiency of MW irradiation in detriment of classical heating by dramatically reducing the reaction time and improving the yield for these two domino cyclocondensation processes, leading to a huge variety of unusual and highly functionalized tricyclic derivatives.

**Scheme 27:**
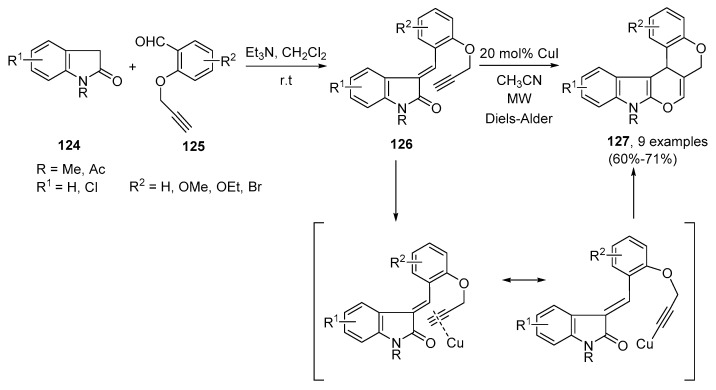
One-pot domino Knoevenagel-hetero-Diels-Alder reactions of dihydropyrano[3,4-*c*]chromenes.

The domino Knoevenagel-hetero-Diels-Alder process is a very efficient tool for one-pot syntheses of polyheterocyclic compounds such as dihydropyrans. In this context, Jha *et al.* [126] reported an efficient two-step synthesis of indole-annulated dihydropyrano[3,4-*c*]chromene derivatives **127** via the Knoevenagel condensation of indolin-2-ones **124** with *O*-propargylated salicylaldehyde derivatives **125**, followed by a microwave-assisted intramolecular hetero-Diels–Alder reaction of the resulting (*Z*)-3-(2-(prop-2-ynyloxy)benzyl-idene)indolin-2-ones **126** in the presence of CuI (20 mol %) (Scheme 27).

In this latter step, the unreactive terminal alkyne is activated by copper through the formation of a π complex. Importantly, a conventional heating failed to afford the cyclized product, even after 72 h of heating in acetonitrile. However, during the hetero-Diels-Alder reaction, the authors observed the formation of a mixture of fully non-polar side-products, which characterizations were impossible. Importantly, other catalysts, such as mesoporous zirconium phosphate [127], nanocrystalline sulfated-zirconia [128] and trifluoroacetic acid, have also been used in this type of reaction and showed also high catalytic activity [129].

**Scheme 28:**
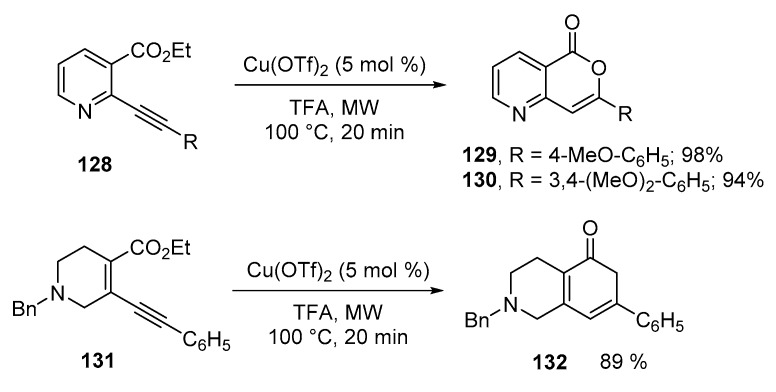
Isocoumarins synthesis under a strong acidic media.

Hellal *et al.* [130] have reported an efficient approach for the synthesis of novel series of isocoumarins under strong acidic conditions using TFA as solvent. The process consisted of a Michael-type (6-*endo*-dig) cyclization of various heterocyclic esters by employing a combination of Brønsted and Lewis acid catalysts. *O*-alkynylaryl esters **128**, **131**, containing nitrogen atoms, were first obtained by means of a Sonogashira coupling reaction and were next cyclized in trifluoroacetic acid (a Brønsted acid) as solvent in the presence of a catalytic amount of Cu(OTf)_2_ (a Lewis acid) under MW-irradiation, leading to a variety of heterocyclic lactones **129**, **130**, **132** in excellent yields. For example, analogues of nicotinate bearing electron-donating groups on the phenyl moiety, led to the corresponding lactones in excellent yields (94%–98%), and the tetrahydropyridine derivative provided the expected product in 89% yield (Scheme 28).

### 2.2. Six-Membered Heterocycles with Two Heteroatoms

#### 2.2.1. Pyridazines

**Figure 9:**
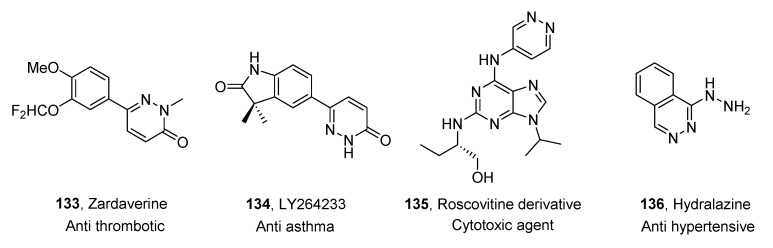
Representative examples of bioactive pyridazines.

Pyridazine is a six-membered ring with two adjacent nitrogen atoms. It is mainly used in medicinal chemistry as building block. The pyridazine structure is found within a number of herbicides and several pharmaceutical drugs (Figure 9) [131,132,133,134,135,136]. Substituted pyridazines have attracted much attention in the fields of organic chemistry for mechanistic investigations [137,138,139,140,141,142,143], as well as in the field of natural products synthesis [140,143,144,145,146].

**Scheme 29:**
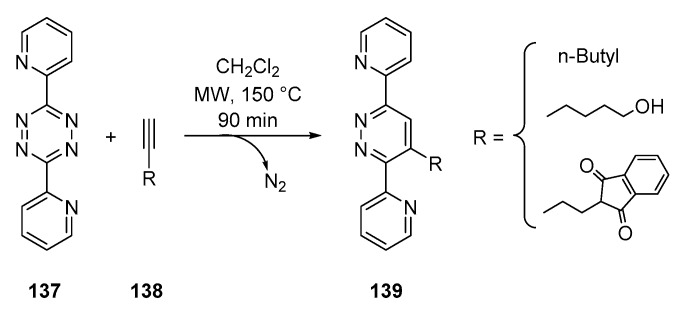
Inverse-electron demand Diels-Alder reaction with alkynes affording mono-substituted 3,6-Di(pyridin-2-yl)pyridazines.

**Scheme 30:**
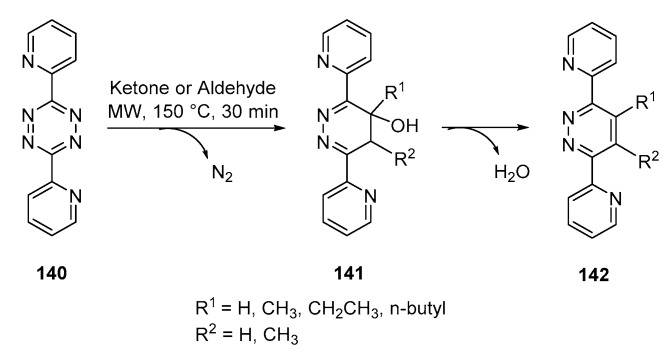
Inverse-electron demand Diels-Alder reaction with ketones or aldehydes affording di-substituted 3,6-Di(pyridin-2-yl)pyridazines.

3,6-Di(pyridin-2-yl)pyridazines are an interesting class of compounds because of their metal-coordinating ability resulting in the self-assembly into [2 × 2] grid-like metal complexes with copper(I) or silver(I) ions. These compounds and other substituted pyridazines can be prepared by inverse-electron demand Diels-Alder reactions between acetylenes and 1,2,4,5-tetrazines. Hoogenboom *et al.* [147] reported that the cycloaddition of 3,6-di(pyridin-2-yl)-1,2,4,5-tetrazine (**137**) with acetylenes **138** could be accelerated from several days in refluxing toluene or DMF to several hours in dichloromethane at 150 °C under MW-activation (Scheme 29). Moreover, they described a novel mechanism for the synthesis of substituted 3,6-di(pyridin-2-yl)pyridazines in which ketones and aldehydes were used as dienophiles in the inverse-electron demand Diels-Alder reaction with tetrazine under MW activation leading to the desired compounds in moderate to good yields (Scheme 30).

**Scheme 31:**
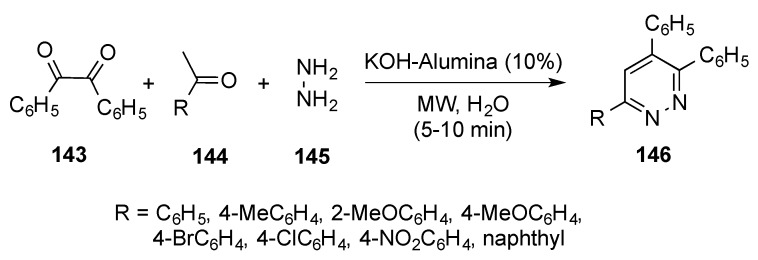
Solvent-free synthesis of 3,4,6-triarylpyridazines using KOH-alumina as catalyst.

Other routes for the synthesis of pyridazine derivatives were described by many scholars [148]. For example a MW-assisted and solvent-free synthesis of 3,4,6-triarylpyridazines **146** and their derivatives has been described by Mecadon *et al.* [149] starting from 1,2-dicarbonyls **143**, active methylene carbonyl species **144**, and hydrazine (**145**) using potassium hydroxide on alumina (KOH-alumina) as a mild, efficient, and recyclable catalyst (Scheme 31). This method led to the expected pyridazines with high yields (73%–89%) after only a few minutes.

#### 2.2.2. Pyrimidines, Quinazolines

**Figure 10:**
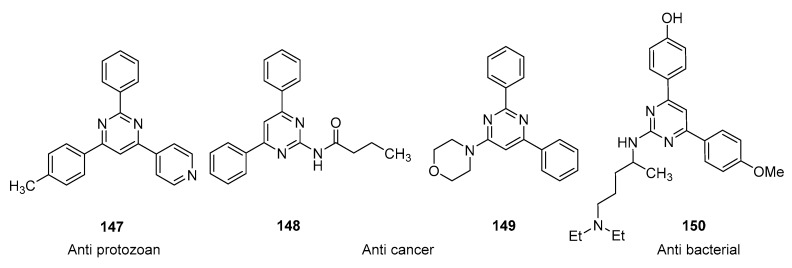
Representative examples of bioactive pyrimidines.

Pyrimidine is a six-membered ring containing two nitrogen atoms at positions 1 and 3 of the heterocycle. Pyrimidine derivatives usually exhibit a huge variety of biological activities, and could be used in therapy as anti-protozoan agents (e.g., **147** exhibits *in vitro* an MIC value of 0.25 µg/mL against *Plasmodium falciparum* [150]), anti-cancer agents (e.g., **148** is a selective adenosine A1 receptor antagonist [151] and **149** is a phosphotidylinositol C kinase inhibitor) [152], or as antibacterial agents (e.g., **150** shows antitubercular activity against *Mycobacterium tuberculosis* at the concentration of 12.5 μg/mL) (Figure 10) [153].

**Scheme 32:**
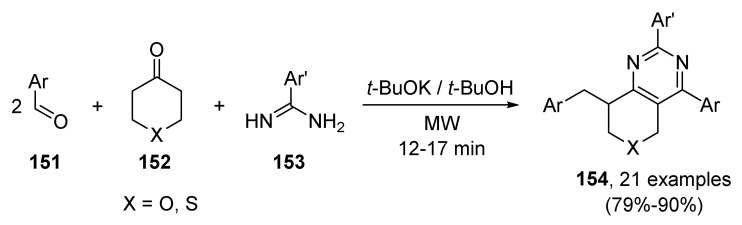
One-pot four-component reaction leading to thiopyran- and pyrano[4,3-*d*]pyrimidines.

Novel synthetic strategies using MW-assisted solution-phase synthesis [154,155] and/or MW-assisted solvent-free synthesis [152,153,154,155] have been extensively used in the last decade for the synthesis of such derivatives. In this context, Jiang *et al.* [156] have described a new synthetic route for the preparation of highly functionalized thiopyrano-, pyrano[4,3-*d*]pyrimidine derivatives incorporating a benzylic core at position 8 of a fused pyran (or thiopyran) nucleus regiospecifically. This synthesis was achieved by using readily available aromatic aldehydes **151**, tetrahydropyran-4-one (or tetrahydrothiopyran-4-one, **152**), and aryl amidines **153** under MW irradiation through a one-pot four-component reaction (Scheme 32). The expected thiopyran- and pyrano[4,3-*d*]pyrimidines **154** have been obtained in good to excellent yields and short reaction times.

**Figure 11:**
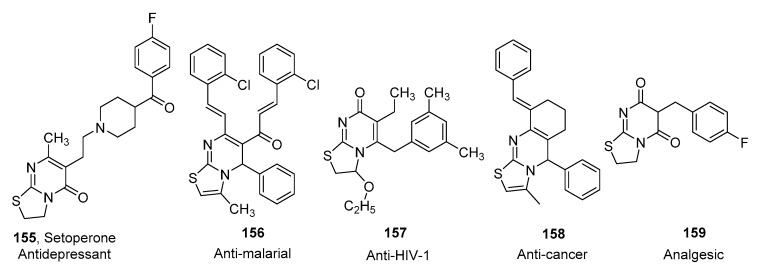
Representative examples of bioactive thiazolo[3,2-*c*]pyrimidines.

Several therapeutic agents contain thiazolo[3,2-*c*]pyrimidine motives, as shown in Figure 11. They possess a range of biological activities including antituberculotic, anti-tumour, bronchodilators, antidepressants, analgesic, anti-human immunodeficiency virus (HIV)-1, anti-inflammatory, anti-microbial and anti-diuretic effects.

**Scheme 33:**
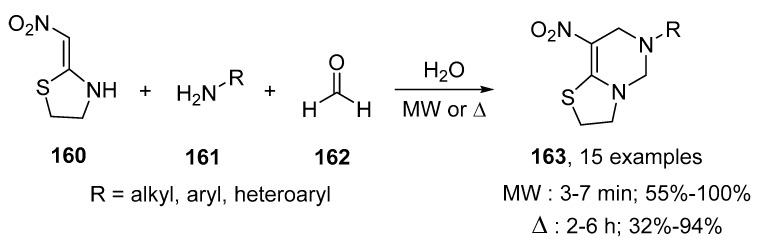
Green syntheses of thiazolo[3,2-*c*]pyrimidines *via* a Manninch reaction.

This prompted Yildirim *et al.* [157] to develop a green procedure leading to new series of these derivatives through Mannich reactions under MW-irradiation. This method involved a multicomponent cyclization of 2-(nitromethylene)thiazolidine (**160**), various aliphatic or aromatic amines **161** and formaldehyde (**162**) in water (Scheme 33). The use of MW activation improved the product yields and significantly shortened the reaction times related to a conventional heating. This green cyclization protocol required neither organic solvent for the reaction or purification of final compounds [158,159,160].

**Scheme 34:**
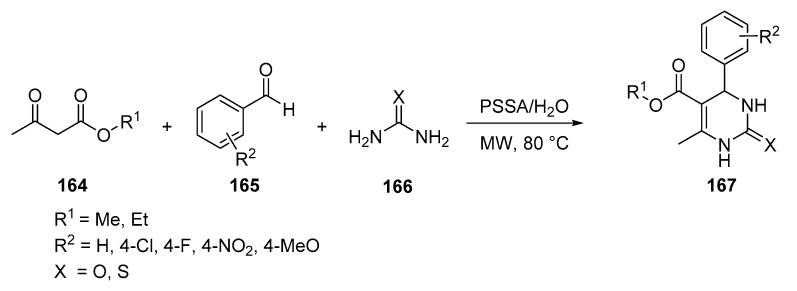
Eco-friendly synthesis of 3,4-dihydropyrimidin-2(1*H*)-ones using polystyrenesulfonic acid in aqueous media.

An elegant protocol for the synthesis of substituted 3,4-dihydropyrimidin-2(1*H*)-ones **167** under MW-irradiation has been highlighted by Varma *et al.* [161]. This involves the condensation of an acetoacetate **164**, substituted benzaldehydes **165** and urea or thiourea **166**, using polystyrenesulfonic acid (PSSA). The reaction occurs efficiently in water without addition of organic solvent (Scheme 34). The use of polymer supported, low toxic and inexpensive PSSA as a catalyst has rendered this method eco-friendly, with a very simple isolation procedure by filtration of the precipitated products.

**Scheme 35:**
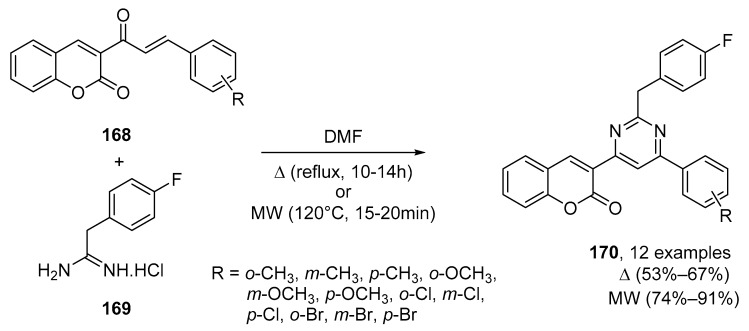
MW-assisted synthesis of fluorinated coumarin-pyrimidine hybrid molecules with promising cytotoxic activites against A-549 and MDA-MB-231 cancer cell lines.

Hosamani *et al.* [162] recently compared MW-assisted and conventional syntheses of a new series of fluorinated coumarin-pyrimidine hybrids. The reactions proceeded through the cyclic-condensation of substituted chalconated coumarins **168** with 2-(4-fluorophenyl) acetamidine hydrochloride (**169**) in DMF, leading to the 3-(2-(4-fluorobenzyl)-4-(substituted phenyl) pyrimidin-6-yl)-2*H*-chromen-2-ones **170**. Improved yields and shortened reaction times have been observed when using MW-irradiation compared to conventional heating (Scheme 35). These newly-synthesized compounds were evaluated *in vitro* for their cytotoxicity against human lung carcinoma cell line A-549 and against the aggressive human breast adenocarcinoma cell line MDA-MB-231, and compared to these of cisplatin, a reference compound used clinically in cancer treatment. In this assay, several molecules exhibited significant cytotoxicity against these two cancer cell lines as underlined by their IC_50_ values, which are below 10 μM. Moreover, the most active compounds exhibited a cytotoxicity against A-549 cell line close to these of cisplatin, IC_50_ = 1.89 μM and are more potent than this reference drug against the MDA-MB-231 cell line.

**Scheme 36:**
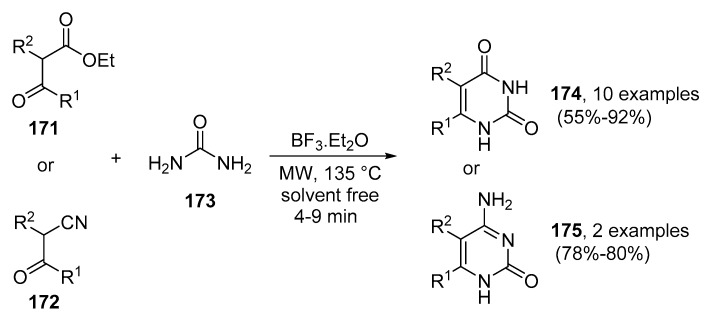
Green synthesis of uracil and cytosine analogues.

MW-assisted solvent-free synthesis has been widely employed for the preparation of pyrimidine derivatives [163,164,165,166,167]. Thus, Burgula *et al.* [166] have reported a green procedure for the single-step preparation of series of uracil and cytosine nucleobases **174** and **175.** Uracil analogues were synthesized by treatment of the respective β-ketoesters or β-aldehydoester **171** with urea, whereas the cytosine derivatives were obtained from benzoylacetonitriles **172** or *N*,*N*-diethylamide precursors (Scheme 36). For these syntheses, using BF_3_·Et_2_O as Lewis acid significantly enhanced the reaction yields as well as the conversion rates.

**Scheme 37:**
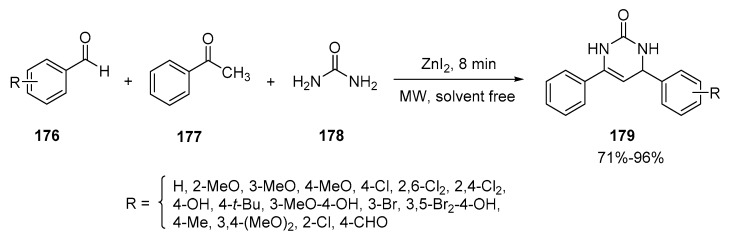
Solvent-free Biginelli multicomponent reaction leading to dihydropyrimidinones.

MW-assisted solvent-free conditions have been also used for a Biginelli multicomponent reaction as illustrated by the synthesis of a new series of 5-unsubstituted-3,4-dihydropyrimidin-2(*1H*)-ones **179** [167]. The procedure involved a three-component, one-pot condensation of an aromatic aldehyde **176**, acetophenone (**177**) and urea (**178**), using ZnI_2_ as catalyst (Scheme 37). This procedure not only provides improved yields related to the conventional Biginelli conditions, but also expands the scope of this process to a larger variety of substrates.

**Figure 12:**
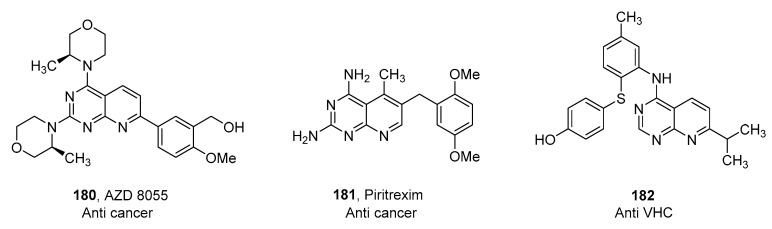
Representative examples of bioactive pyridopyrimidines.

The pyridopyrimidine motive is found in a large variety of bioactive substances including several marketed drugs. For example, this system is present in AZD8055 (**180**), a selective ATP-competitive PI3KAkt-mTOR signaling pathway inhibitor [168], piritrexim (**181**), a lipid-soluble inhibitor of dihydrofolate reductase (DHFR) that displays high potency for the treatment of metastatic urothelial cancer [169], and pyrido[2,3-*d*]pyrimidine **182** that is a hepatitis C virus inhibitor [170] (Figure 12). Other pyridopyrimidine derivatives have also been reported for their potential use as anticardiovascular [171], anti-inflammatory [172,173], antibacterial [174,175], and anti-Parkinson agents [176].

**Scheme 38:**
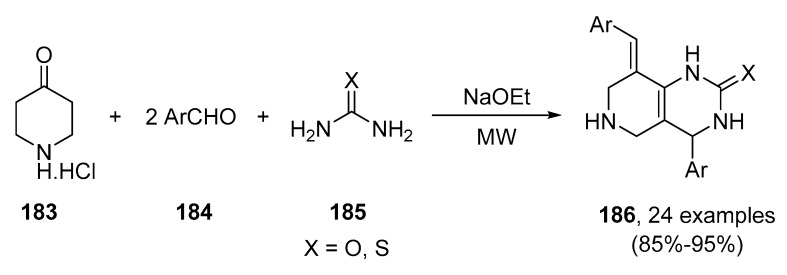
One-pot, solvent free, four-component procedure affording pyridopyrimidines.

**Scheme 39:**
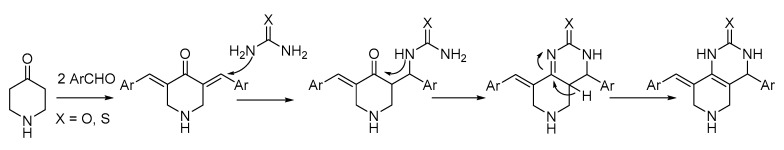
Mechanistic study of the pyridopyrimidines synthesis reported by Basiri *et al.* [177].

In this context, novel series of pyridopyrimidines were synthesized by Basiri *et al.* [177] through a one pot four-component MW-assisted reaction. This procedure, under solvent-free condition, involved 4-piperidone hydrochloride monohydrate (**183**), aromatic aldehydes **184** and urea or thiourea **185**, in the presence of catalytic amount of solid sodium ethoxide (Scheme 38). Interestingly, the reactions were completed in 30 seconds only, affording the expected pyridopyrimidines **186** in excellent yields and with a high degree of purity. This reaction proceeds through a domino sequence involving Michael addition, condensation and tautomerization, as outlined in Scheme 39.

The newly synthesized pyridopyrimidine derivatives were evaluated *in vitro* for their potencies as inhibitors of the acetyl-cholinesterase (AChE) and the butyrylcholinesterase (BChE). Among them, two compounds are 2.5 times (IC_50_ = 0.8 µM) and 1.5 times (IC_50_ = 1.37 µM) more potent in inhibiting AChE than galanthamine, which is the reference inhibitor for these enzymes. It is noteworthy that pyrimidinone derivatives are better AChE inhibitors than pyrimidinthiones. In addition, these newly synthesized pyridopyrimidines derivatives exhibited IC_50_ values ranging from 1.18 to 18.90 μM in BChE inhibition, and therefore displayed better activities than the reference drug. The structure-activity relationship study underlines that the unsubstituted and *ortho*-substituted derivatives are more potent BChE inhibitors than their *meta*-, *para*-, and disubstituted analogues.

**Figure 13:**
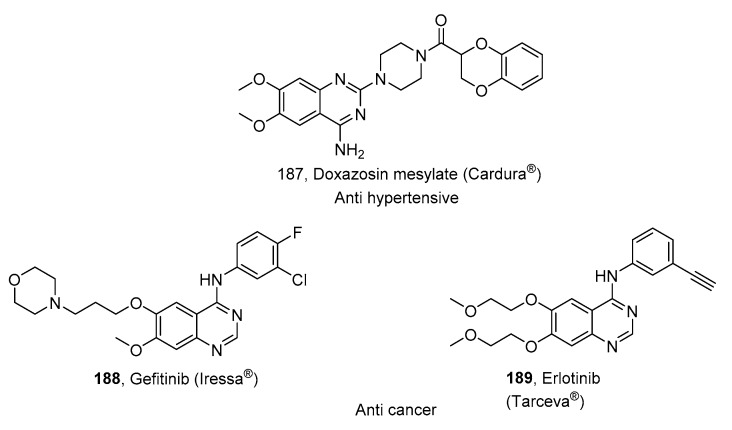
Representative examples of bioactive quinazolines.

Quinazoline derivatives are commonly used in medicinal chemistry as anti-malarial and anti-cancer agents [178,179,180,181,182,183]. Doxazosin mesylate (**187**, Cardura^®^), a drug used in the treatment of hypertension [184], gefitinib (**188**, Iressa^®^) and erlotinib (**189**, Tarceva^®^) are epidermal growth factor receptor inhibitors approved for the treatment of lung cancer and are relevant examples of marketed drugs encompassing such heterocyclic motive [185,186] (Figure 13).

**Scheme 40:**
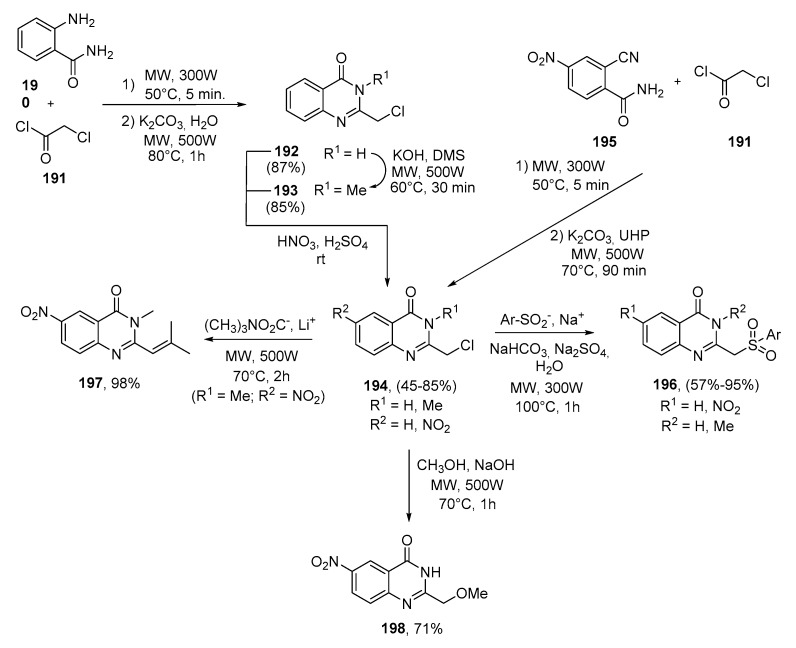
MW-assisted synthesis of 2-substituted quinazolines.

In the last decade, numerous syntheses of highly functionalized series of quinazoline derivatives have been described in the literature [187,188,189,190,191,192,193,194,195,196,197,198,199]. Kabri *et al.* [187] reported several studies devoted to the synthesis of 2-substituted quinazolines (Scheme 40). The key intermediates for this synthesis were easily obtained through green procedures. On the one hand, 2-aminobenzamide (**190**) reacted under MW-activation with chloroacetylchloride (**191**) to afford *N*-(2-aminophenyl)-2-chloroacetamide, an intermediate which could be cyclized into 2-chloromethylquinazoline-4(3*H*)-one (**192**) in basic aqueous media and under MW activation. The treatment of **192** under MW-activation by dimethylsulfate in a mixture of water and THF leads to the corresponding *N*-methylated derivative **193**. The nitration of **192** or **193** using a mixture of HNO_3_ and H_2_SO_4_ leads regioselectively to 3-methyl-6-nitroquinazolin-4(3*H*)-one derivatives **194**. On the other hand, to avoid the last nitration step, the authors described an alternative route consisting in the use of 2-methyl-5-nitrobenzonitrile (**195**) as starting material.

These key intermediates **194** have been functionalized at position 2 according to different green procedures. On the one hand, these derivatives have been submitted to *S*-alkylations using sodium salts of various benzenesulfinic acid as alkylating agents. These syntheses occurred in aqueous media via a SN_2_ mechanism to afford the quinazoline series **196**; the MW-irradiation improved drastically conversion rates and reaction times related to conventional conditions. On the other hand, ethylenic derivatives **197** have been obtained by applying MW-activation to a radical S_RN_1 reaction. To this end, **194** derivatives have been treated by a series of nitropropane anions. The resulting *C*-alkylated intermediate is unstable, and nitrous acid is eliminated rapidly in basic medium, due to the acidity of the methylene protons, resulting in the formation of **197**. Under microwave irradiation, this conversion occurs quantitatively, in 15 min. This reaction has been extended to a series of various nitronate anions. Otherwise, the substitution of the chlorine by a methoxy group using MeOH in a basic medium leads to compound **198** with 71% yield even under MW-activation.

**Scheme 41:**
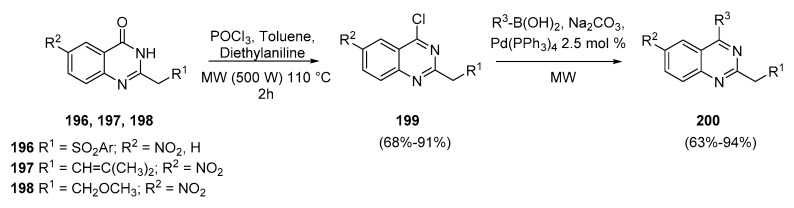
Quinazolinones functionalization through a MW-assisted Suzuki-Miyaura cross-coupling reaction.

The functionalized quinazolinones **196**, **197** and **198** have been chlorinated using POCl_3_ through a MW-assisted chlorination procedure, affording the corresponding **199** derivatives in 70%–90% yield (Scheme 41). Then, these intermediates have been used as substrates in Suzuki-Miyaura cross-coupling reactions, to be functionalized in position 4 (compound **200**). The use of MW-irradiation allowed significant improvements in terms of reaction times and conversion rates; unfortunately, these reactions were not compatible with aqueous media.

**Scheme 42:**
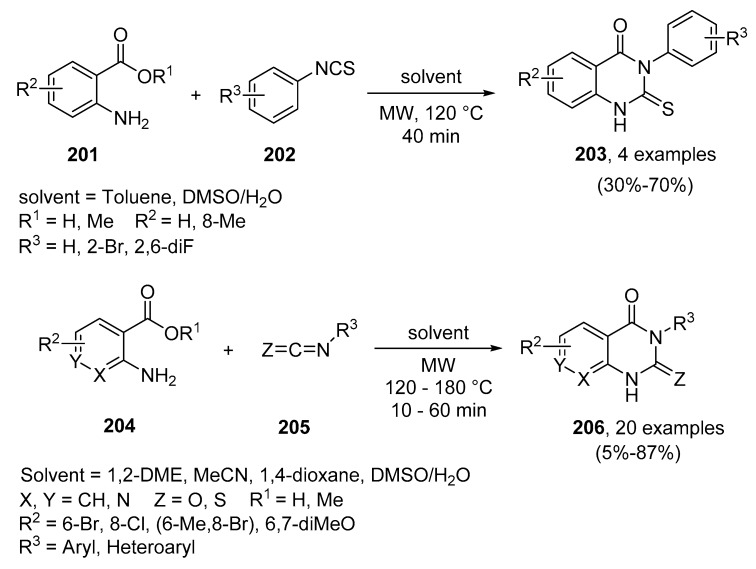
MW-assisted synthesis of 2-thiooxoquinazolinone with potential application in stroke treatment.

In a search for new phosphodiesterases inhibitors with potential application in stroke treatment, Redondo *et al.* [195] have reported new syntheses of new 2-thiooxoquinazolinone derivatives (**203**, **206**, Scheme 42). These compounds were easily obtained under MW-irradiation by cyclocondensation reactions involving functionalized anthranilic acid derivatives **201**, **204** and isocyanates or isothiocyanates **202**, **205**.

These newly synthesized compounds exhibit an overall anti-inflammatory potency, and they exert a neuroprotective activity as demonstrated by the reduction of the nitrite production in a primary neural cells culture model. In addition, in a permanent middle cerebral artery occlusion stroke model, one of these molecules improved the behavioral outcome.

**Scheme 43:**
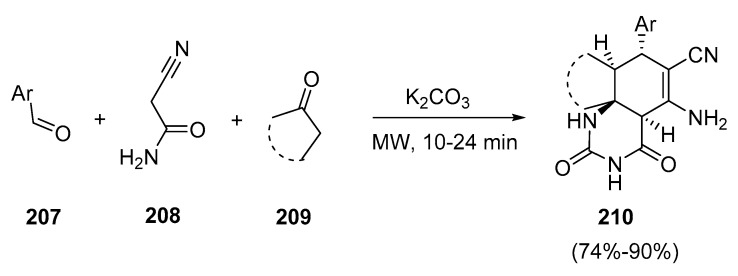
Four-component domino reactions affording pyrido[3,4-*i*]quinazolines.

A new multi-functionalized quinazoline synthesis through a four-component domino reaction has been described by Jiang *et al.* [196]. A series of pyrido[3,4-*i*]quinazoline derivatives **210** were synthesized in good to excellent yields by MW-irradiation from a mixture of aromatic aldehydes **207**, various cyclic carbonyl compounds **208** and cyanoacetamide (**209**) in ethylene glycol using K_2_CO_3_ as base (Scheme 43). The reactions were completed in only 10–24 min, and water was the major byproduct, making workup and purification very convenient.

**Scheme 44:**
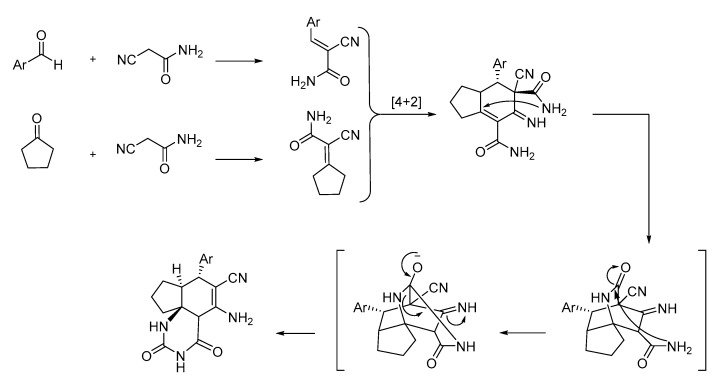
Mechanistic study of the pyrido[3,4-*i*]quinazoline synthesis reported by Jiang *et al.* [196].

The authors hypothesized, as a key step for the domino reaction, the tandem formation of two Knoevenagel intermediates, and their subsequent [4 + 2] cycloaddition, as outlined in Scheme 44. This new MW-assisted process allowed exceptional chemo-, regio- and stereoselectivity of this synthesis, as illustrated by the excellent control of the stereochemistry of the four stereogenic centers of the final quinazoline. Importantly, the X-ray crystal analyses, combined with NMR studies, allowed the unambiguous determination of the absolute configuration of the final compounds.

**Scheme 45:**
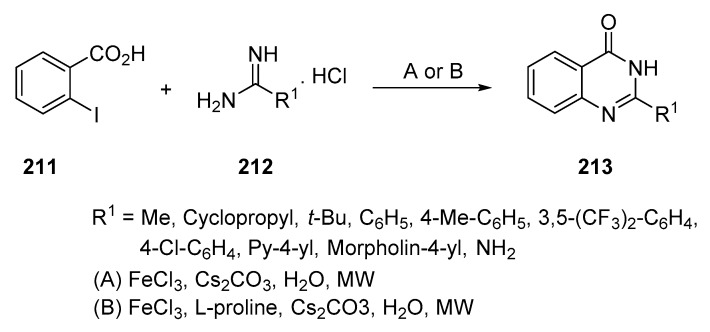
First example of iron-catalyzed C–N coupling in aqueous media leading to quinazolinones.

Zhang *et al.* [197] have reported the first example of iron-catalyzed C–N coupling reactions in aqueous media to afford *N*-heterocycles. This procedure has been applied for the green synthesis of quinazolinone derivatives **213** from substituted 2-halobenzoic acids **211** and amidines **212** via MW-activation. The cyclization reaction is catalyzed by iron chloride in the presence or not of a ligand such as L-proline in water (Scheme 45). The expected products were obtained with moderated to high yields, even with inactive substrates such as guanidines.

**Scheme 46:**
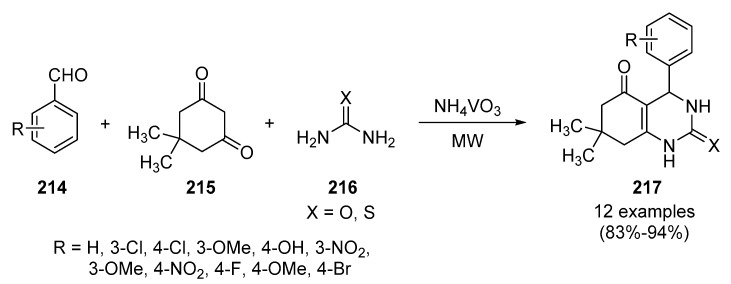
MCR reaction leading to octahydroquinazolinones using ammonium metavanadate as catalyst.

A simple and green one-pot synthesis of octahydroquinazolinone derivatives under solvent-free MW-assisted conditions has been described Niralwad *et al.* [198]. The reaction was performed by adding substituted benzaldehydes **214**, dimedone (**215**) and urea (or thiourea) **216** in the presence of ammonium metavanadate as catalyst (Scheme 46). The procedure afforded the desired octahydro-quinazolinone derivatives **217** in high yields after a short reaction time.

**Scheme 47:**
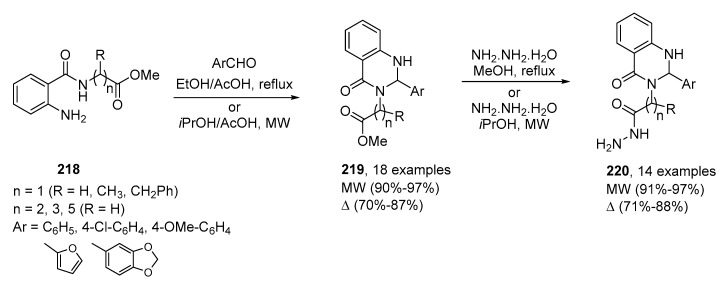
MW-assisted synthesis of quinazolinones with a potential activity as MAO inhibitiors.

A novel series of quinazolinone amino acid ester and quinazolinone amino acid hydrazides were prepared by Khattab *et al.* [199] under MW-irradiation and compared with conventional conditions. For example, the reaction of methyl (2-aminobenzamido) ester derivatives **218** with different aldehydes under conventional conditions (reflux for 8 h) or by using MW irradiation (10 min) afforded the substituted quinazolinone amino acid esters **219**. In a second step, these intermediates reacted with hydrazine hydrate using conventional heating (8 h) or microwave irradiation (10 min) to yield the corresponding hydrazides **220** (Scheme 47). MW-irradiation afforded the expected hydrazides with shortened reaction times and improved yields compared to conventional heating.

Moreover, these compounds have been studied for their activity on monoamine oxidases (MAO). Indeed, three compounds exhibited IC_50_ values in the 2–3.6 nM range against MAO-A which is close to the value obtained with the standard inhibitor, clorgyline (IC_50_ = 2.9 nM). Interestingly, these compounds do not exhibit oral and parental acute toxicity as demonstrated by *in vivo* experiments on male mice. In this assay, the animals tolerated up to 300 mg/Kg of the drug (oral administration) and up to 125 mg/Kg dose by parenteral administration.

#### 2.2.3. Piperazines, Pyrazines and Oxazines

**Figure 14:**
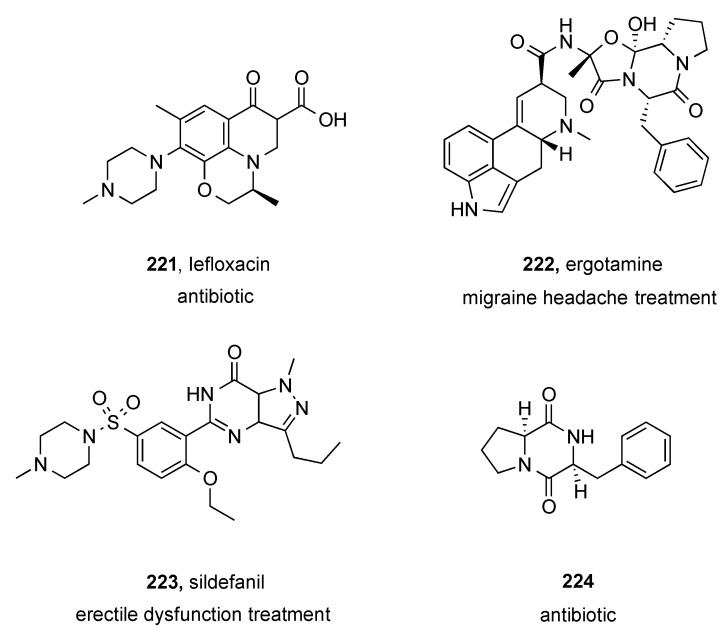
Representative examples of bioactive piperazines.

Many pharmacological drugs feature a piperazine motive. Among them, diketopiperazines and their higher-functionalized analogues, the epithiodiketopiperazines, are common motifs that can be found in several natural products [200,201,202,203]. Overall, 2,5-diketopiperazine derivatives emerged in the last decade as very attractive molecules in drug discovery [204,205,206,207,208,209,210]. Indeed, these derivatives usually exhibit various biological properties [211], including antitumor [212,213] antiviral [214], antifungal [215,216] antibacterial [217,218] or antifouling activities [219]. Interestingly, their role in the bitter taste of coffee, beer, cacao and chocolate has been highlighted [220]. In addition, the use of these diketopiperazines as organic catalysts for the hydrocyanation of imines has been previously reported (Figure 14) [221].

**Scheme 48:**
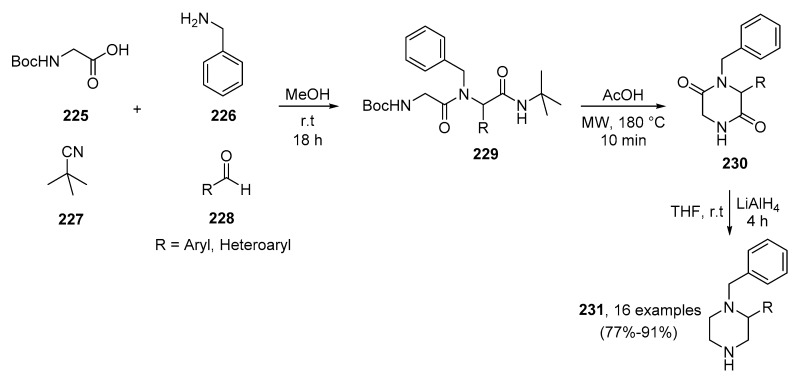
MW-assisted Ugi 4-component reaction leading to piperazines.

A series of diketopiperazines has been synthesized by Jida *et al.* through the four component Ugi procedure in the presence of acetic acid, and under MW-irradiation at 180 °C [204]. Interestingly, the reduction of these molecules using LiAlH_4_ afforded the corresponding functionalized piperazines in good yields (Scheme 48).

**Scheme 49:**
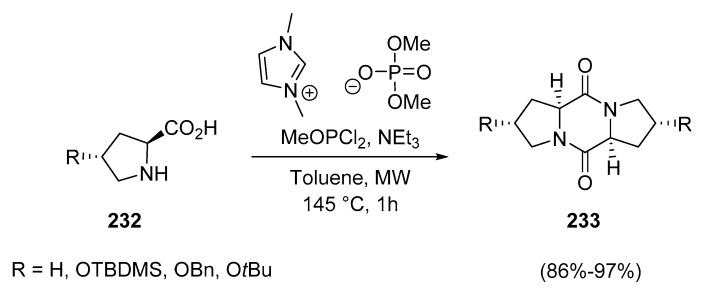
One-pot stereoselective synthesis of 2,5-diketopiperazines.

Jainta *et al.* [205] have described a MW-assisted stereoselective one-pot synthesis of symmetrical and unsymmetrical 2,5-diketopiperazines (Scheme 49). The reaction was carried out via a facile condensation of unprotected amino acids **232** by a phosphite-promoted one-step coupling reaction. This method leads to the expected compounds **233** with good overall yields. This strategy can be used for large-scale synthesis and is compatible with several protecting groups. Importantly, neither racemization nor inversion of the stereochemistry has been reported when chiral starting materials were used. Lastly, the final work-up, which consist on a simple filtration step in order to remove the ammonium salts and ionic liquid is very easy and no further purifications are required. Therefore, the expected 2,5-diketopiperazines were obtained in quantitative yields.

**Scheme 50:**
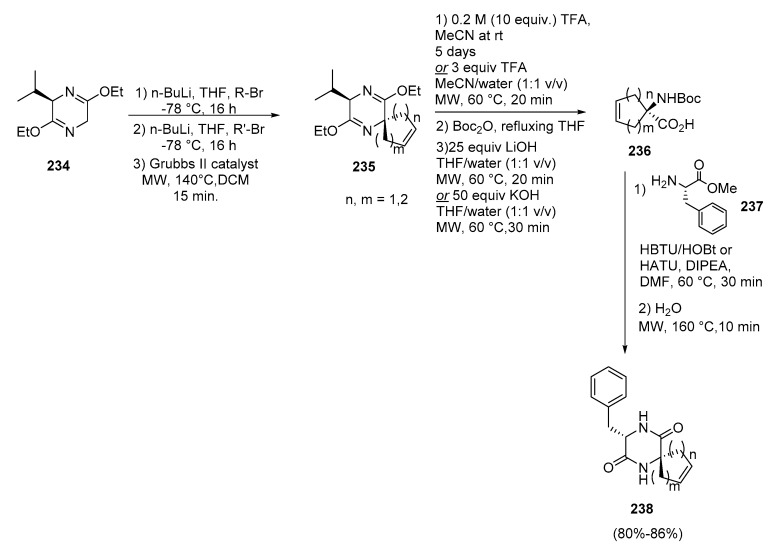
Synthetic route to spiro-fused 2,5-diketopiperazines via sequential Schoellkopf ring-closing metathesis reactions.

A general and efficient method for the synthesis of *spiro*-2,5-diketopiperazines (e.g. **238**, *spiro*-DKPs) has been described by Jam *et al.* [206]. In the first step of this preparation, *spiro*-amino acids were synthesized by combining in the one hand, stereoselective alkylation reactions for amino acid construction (Schoellkopf synthesis) and on the other hand, a Grubbs ring-closing metathesis catalyzed by ruthenium complexes. Next, the Boc-protected dipeptides containing *spiro*-amino acids were cyclized through MW activation in water to afford the corresponding *spiro*-DKPs in high yields and short reaction times (Scheme 50).

**Scheme 51:**
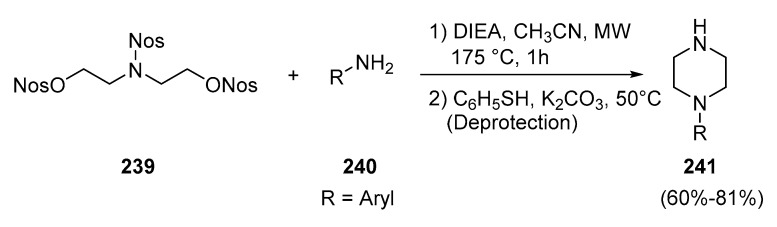
MW-assisted synthesis of sterically hindered *N*-arylpiperazines.

Gao *et al.* [207] have reported practical synthesis of a huge variety of sterically hindered *N*-arylpiperazine derivatives **241**. The reaction proceeded by mixing 2,2′-(4-nitrophenylsulfonyl-azanediyl)bis(ethane-2,1-diyl)bis(4-nitrobenzenesulfonate) (**239**) and substituted anilines **240** under MW-irradiation (Scheme 51). This method can be used in parallel and also in combinatorial chemistry for the preparation of different libraries of highly functionalized piperazines, since the synthesis are completed within short times.

**Figure 15:**
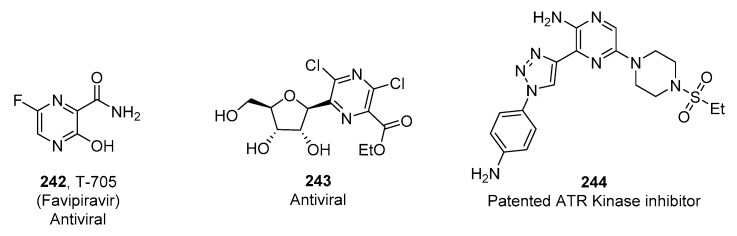
Representative examples of bioactive pyrazines.

Pyrazine is a six membered hetero-aromatic ring usually found in the structure of numerous natural products and synthetic compounds, some of which are used in the food industry for their flavor properties [222,223,224]. Moreover, they are versatile synthetic intermediates [225], and many functionalized pyrazines possess pharmacological activities, such as antiviral [226,227] (**242**, **243**), ATR kinase inhibitor [228] (**244**), antitumor [229], vascular endothelial growth factor inhibitory activity [230], or as epithelial sodium channel blockers [231] (Figure 15).

**Scheme 52:**
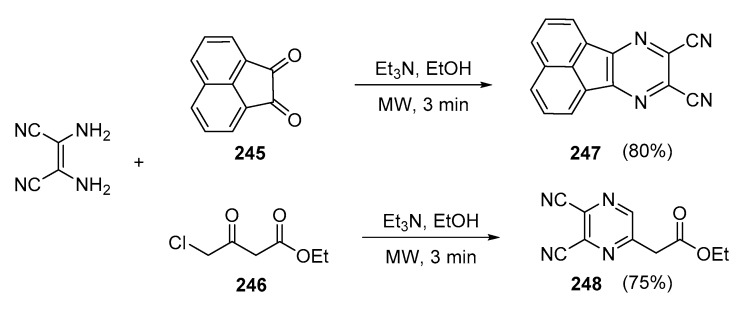
MW-assisted synthesis of dicyanopyrazines.

Original synthetic heterocycles, such as substituted dicyanopyrazines **247**, **248**, have been synthesized using a domestic MW oven [232]. The reaction proceeded by a cyclocondensation of diaminomalonitrile with ketones (e.g., acenaphthenequinone, **245**) or an ester **246** in the presence of triethylamine in ethanol (Scheme 52), and the expected products were obtained with good to excellent yields.

**Scheme 53:**
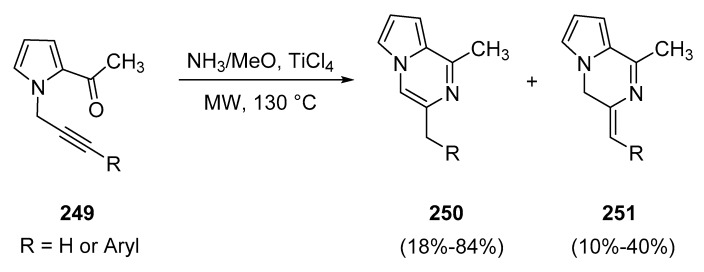
Synthesis of pyrrolo[1, 2-*a*]pyrazines through a imination/annulation tandem reaction.

**Scheme 54:**
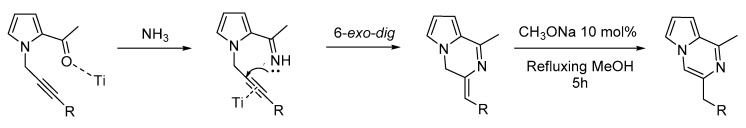
Mechanistic study of the pyrrolo[1, 2-*a*]pyrazines synthesis reported by Alfonsi *et al.* [233].

Moreover, Alfonsi *et al.* [233] have reported the synthesis of pyrazines derivatives and other series of *N*-heterocycles **250**, **251** by tandem imination/annulation of γ- and δ-ketoalkynes **249**. The reaction was achieved by the intramolecular cyclization of 2-acetyl-1-propargylpyrroles in the presence of ammonia under MW irradiation leading mostly to the pyrazine derivatives (Scheme 53). The authors suggest that the reaction proceeds firstly by the formation of the imine, catalyzed by a Lewis acid (TiCl_4_). This intermediate undergoes intramolecular 6-*exo*-*dig* cyclization, by reacting with the triple bond activated by TiCl_4_ (Scheme 54). Then, the resulting 3,4-dihydropyrrolo[1,2-*a*]pyrazine derivatives **251** lead to the thermodynamically more stable pyrrolo[1,2-*a*]pyrazines **250**.

**Scheme 55:**
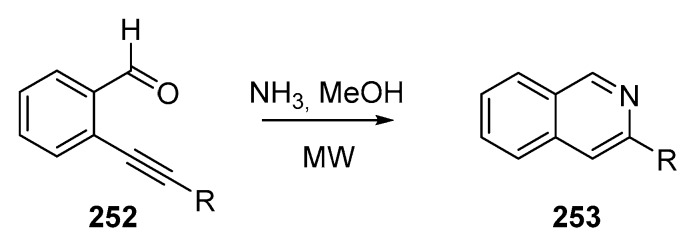
Synthesis of isoquinolines through a imination/annulation tandem reaction.

**Scheme 56:**
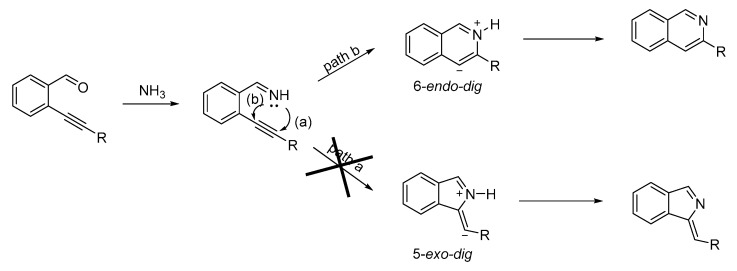
Mechanistic study of the isoquinolines synthesis reported by Alfonsi *et al.* [233]

These results prompted the authors to use also 2-alkynylbenzaldehydes **252** as starting material for this annulation reaction. As anticipated, these compounds afforded the isoquinolines **253** under MW-irradiation and in the presence of ammonia, but no catalyst was required in this case due to the higher reactivity of the aldehyde (Scheme 55). This annulation proceeded regiospecificaly, through a 6-*endo-dig* mechanism. The authors hypothesized a mechanism involving the formation of an imine, subsequent cyclization and then proton shift. They reported a total regioselectivity for the cyclization, since they never observed the formation of any 5-*exo-dig* cyclization adduct. They demonstrated that this selectivity could be attributed to a higher thermodynamic stability of the zwitterionic intermediate resulting from the 6-*endo-dig* mechanism (Scheme 56).

**Scheme 57:**
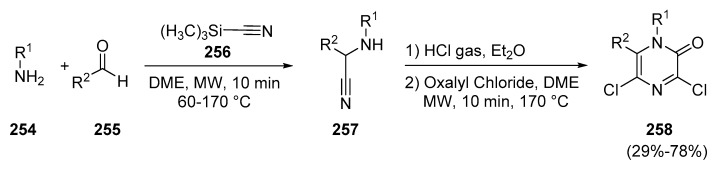
Two-step synthesis of 2(1*H*)-pyrazinones.

Derivatives of 2(1*H*)-pyrazinones are considered as important scaffolds for drug design due to their widespread biological activities [234,235,236,237,238,239]. In this context, Gising *et al.* [237] have reported a rapid and versatile one-pot MW-assisted two steps synthesis of *N*-1 and *C*-6 functionalized 3,5-dichloro-2(1*H*)-pyrazinones **258**. In the first step, the reaction of a primary amine **254**, an aldehyde **255** and trimethylsilyl cyanide (**256**) in 1,2-dimethoxyethane under MW-irradiation leads to substituted α-aminonitriles **257**. In the second step, the expected pyrazinones are obtained through the cyclocondensation of the α-aminonitrile with oxalyl chloride in the presence of gaseous HCl in 1,2-dimethoxyethane; this second step occurs also under MW-irradiation (Scheme 57).

**Scheme 58:**
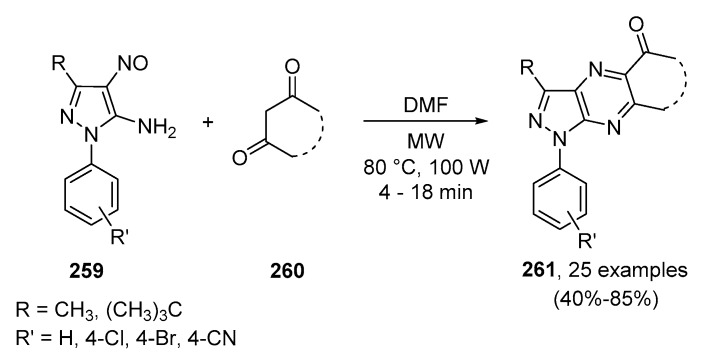
MW-assisted Ehrlich-Sachs type reaction leading to pyrazolo[3,4-*b*]pyrazines.

**Scheme 59:**
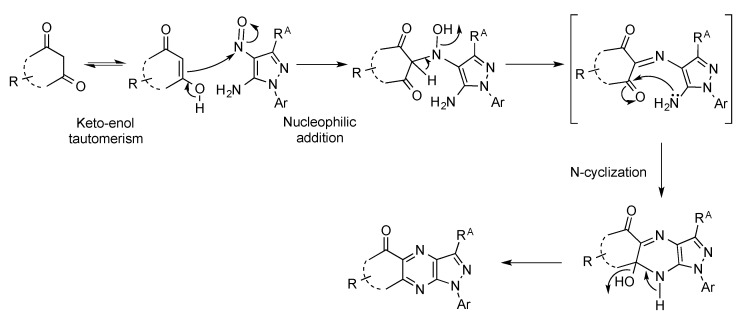
Mechanistic study of the pyrazolo[3,4-*b*]pyrazines synthesis reported by Quiroga *et al.* [239].

An original synthetic route to pyrazolo[3,4-*b*]pyrazine derivatives **261** has been described by Quiroga *et al.* [239], which proceeds through a MW-induced cyclocondensation of *ortho*-aminonitrosopyrazoles **259** and cyclic β-diketones **260** in DMF through an extension of the Ehrlich-Sachs reaction (Scheme 58). According to the authors’ hypothesis, the reaction is initiated by chemo-selective addition of the enol tautomer of the diketone to the nitroso group of the pyrazole, resulting in formation of an imine. This intermediate undergoes an intramolecular *N*-cyclization involving the amino group (NH_2_) of the pyrazole and the remaining carbonyl group (Scheme 59). The compounds were isolated in moderate to good yields, and easily purified *via* recrystallizations. The use of classical heating instead of MW required longer reaction times and led to lower conversion rates.

**Figure 16:**
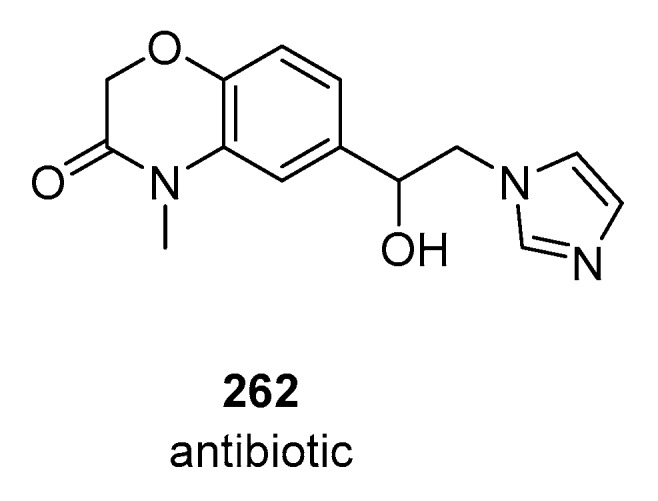
A typical example of bioactive oxazine.

Oxazine derivatives are also considered as important pharmacophores in drug discovery (Figure 16). Indeed, a large number of natural and synthetic bioactive compounds contain a 2*H*-1,4-benzoxazine scaffold [240]. For instance, the enediyne antitumor antibiotic, C-1027 [241] consists of a 2-methylene-3,4-dihydro-3-oxo-2*H*-1,4-benzoxazine moiety in the chromophore subunit. Many derivatives of 2*H*-1,4-benzoxazine have been reported as plant resistance factors against insects and also microbial diseases [242], serotonin-3 (5-HT3) receptor antagonists [243,244,245], potassium channel modulators [246], *etc.* The 3,4-dihydro-3-oxo-2*H*-1,4-benzoxazine scaffold is also considered a bioisoster for 2(3*H*)-benzoxazolone [247] and is used as a privileged scaffold in drug design. Benzo[1,4]oxazin-3-one derivatives also exhibit multiple biological activities, such as anti-inflammatory [248], antiulcer [249], antipyretic [250], antihypertensive [251], and antifungal properties [252]. Some of them also act as 5-HT ligands [253], DP receptor antagonists [254] and integrin antagonists [255]. Therefore, the benzo[1,4]-oxazin-3-one scaffold can be pointed out as a very “privileged pharmacophore” [256,257,258].

**Scheme 60:**
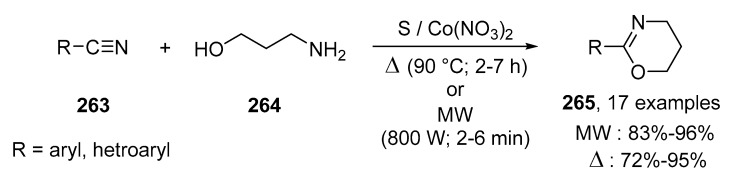
Two-component solvent-free synthesis of 2-oxazines.

A two-component solvent-free procedure for the synthesis of 2-oxazines **265** under thermal and MW conditions has been described by Ge *et al.* [259]. Thus, mono- and bisoxazines were selectively synthesized in good to excellent yields and with shortened reaction times (Scheme 60). The authors have improved the reaction by using a combination of sulfur along with Co(NO_3_)_2_ as the catalytic system. In addition, the catalyst can be reused at least seven times without significant loss of activity.

**Scheme 61:**
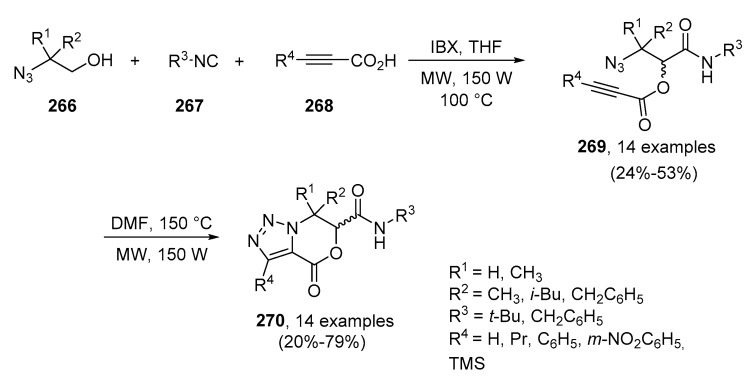
Two-step synthesis of triazolo-fused oxazinones *via* a Passerini cycloaddition process.

De Moliner *et al.* [260] reported the synthesis of novel series of triazolo-fused oxazinones **270**, in a two step procedure. The synthesis occurs through a MW-assisted Passerini cycloaddition process. In this protocol, the authors have obtained the three-component adducts in moderate to good yields, by reacting α-azido aldehydes, generated *in situ* by oxidation of corresponding α-azido alcohols with iodobenzoïc acid (IBX, **266**) with isocyanides **267** and various propiolic acids **268** (Scheme 61). The triazolo-fused dihydro-oxazinones have been obtained as an equimolar mixture of diastereoisomers through a straightforward azide-alkyne dipolar cycloaddition.

**Scheme 62:**
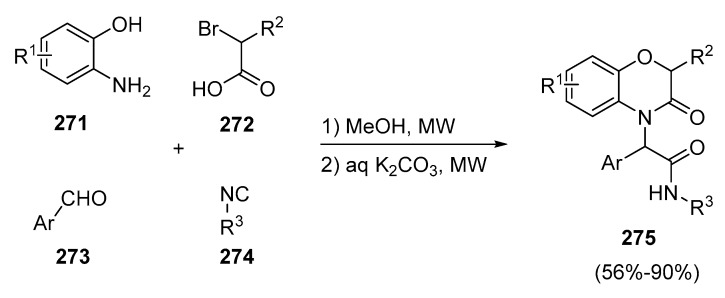
One-pot, Ugi four-component reaction affording 3,4-dihydro-3-oxo-2*H*-1,4-benzoxazines.

**Scheme 63:**
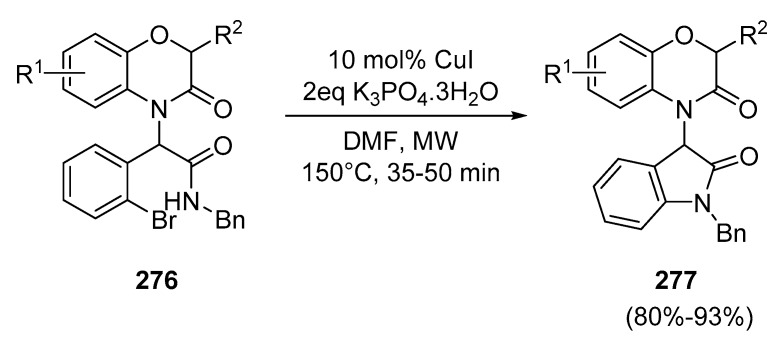
Functionalization of 3,4-dihydro-3-oxo-2*H*-1,4-benzoxazines through a MW-assisted CuI catalyzed intramolecular amidation.

Xing *et al.* [261] have reported a MW-assisted one-pot synthesis of highly functionalized 3,4-dihydro-3-oxo-2*H*-1,4-benzoxazines **275**. The reaction proceeded *via* an Ugi four-component reaction and intramolecular *O*-alkylation sequence of 2-aminophenols **271**, aromatic aldehydes **273**, α-bromoalkanoic acids **272** and isocyanides **274**, using K_2_CO_3_ in methanol (Scheme 62). Further post-modification has been showcased by the MW-assisted CuI catalyzed intramolecular amidation within 3,4-dihydro-3-oxo-2*H*-1,4-benzoxazines **276** affording an original heterocyclic scaffold **277** (Scheme 63).

### 2.3. Six-Membered Heterocycles with Three or Four Nitrogen: Triazines and Tetrazines

**Figure 17:**
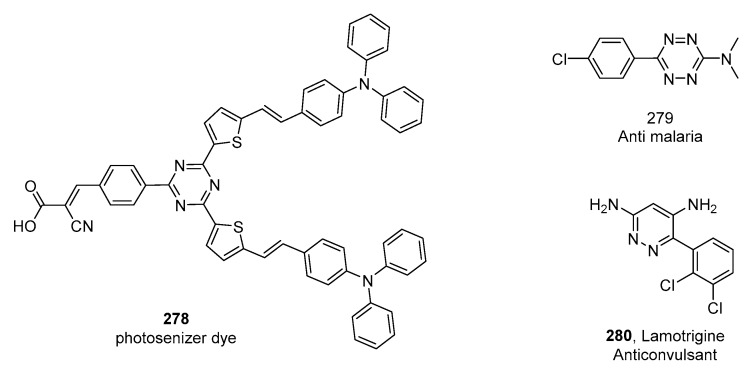
Representative examples of bioactive triazines and tetrazines.

The π-deficient triazines [262] and tetrazines [262,263] are relevant building blocks for more complex molecules as such heterocycles can be used as reactants in LUMO diene-controlled [4+2] inverse electron demand Diels–Alder cycloaddition processes, which efficiently lead to substituted dihydropyridazines, pyridazines, pyrimidines, or pyridine derivatives [264]. The pioneering work dealing with this so-called Carboni–Lindsey reaction has been reviewed by Boger [265]. Triazine and tetrazine derivatives are commonly used as commercial dyes [266], as insecticides [267] and more recently as pharmaceutical agents (Figure 17) [268,269]. Many examples of Diels–Alder reactions leading to new series of triazines and tetrazines have been described in the last decade [262], and among them several proceeded through a MW-assisted procedure. In marked contrast to the triazine derivatives, which have been extensively reviewed in recent years, MW-assisted syntheses of tetrazines remain more underreported, and this review has allowed us to highlight some representative preparations of this system.

**Scheme 64:**
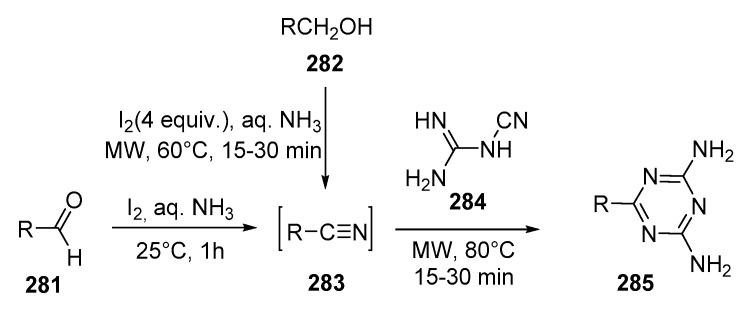
One-pot tandem reaction in aqueous media leading to 1,3,5-triazines.

A practical MW-assisted method for the direct transformation of aldehydes **281** and primary alcohols **282** into triazines in aqueous media was described by Shie *et al.* [270] The alcohols and aldehydes reacted with iodine in aqueous ammonia to provide the corresponding nitrile intermediates **283**, which readily underwent [2+3] cycloadditions with dicyandiamide (**284**) under MW-irradiation at approximately 80 °C for 15–30 min to give the corresponding 4-aryl-2,6-diamino-1,3,5 triazines **285** in 69%–83% yields (Scheme 64). This method circumvents the problem of prior preparation of nitrile compounds from halides and toxic cyanides. The one-pot tandem reactions were conducted in aqueous media, and the products were obtained simply by filtration. In comparison with the previously reported heating methods, microwave irradiation has an advantage in shortening reaction times and increasing the transformation yields.

**Scheme 65:**
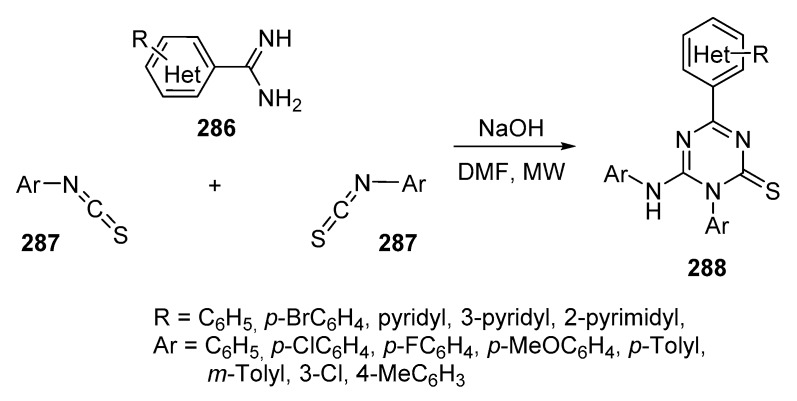
Base-promoted one-pot domino [3+2+1] heterocyclizations to 1,3,5-triazines.

Recently, Li *et al.* [271] have developed an efficient method for the synthesis of a series of *N*-arylamino substituted 1,3,5-triazines, which consist on a base-promoted domino [3+2+1] heterocyclization between aromatic isothiocyanates **286** and aryl amidines **287** in the presence of NaOH under MW-activation. The synthesis showed attractive properties such as concise one-pot conditions, short reaction times (15–24 min), and easy purification of the crude material. In addition, final 1,3,5-triazine derivatives **288** have been obtained with good yields (60%–78%) (Scheme 65).

**Scheme 66:**
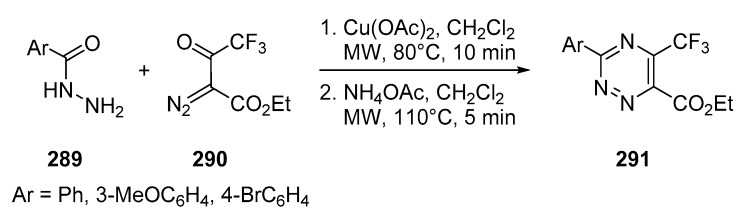
Two-step synthesis of 1,2,4-triazines.

Moreover, Moody and co-workers [272,273] have reported the synthesis of 1,2,4-triazines using a new two-step sequence for the conversion of hydrazides **289** to triazines **291**. The key steps are N–H insertion by a copper carbene intermediate derived from α-diazo-β-ketoesters **290** into the hydrazide, followed by reaction with ammonium acetate to give 1,2,4-triazines. The authors showed that the use of copper(II) combined with MW-irradiation accelerate the reaction. A diverse set of trifluoromethyl-1,2,4-triazines **291** have been obtained in moderate to good yields (28%–91%) (Scheme 66).

**Scheme 67:**
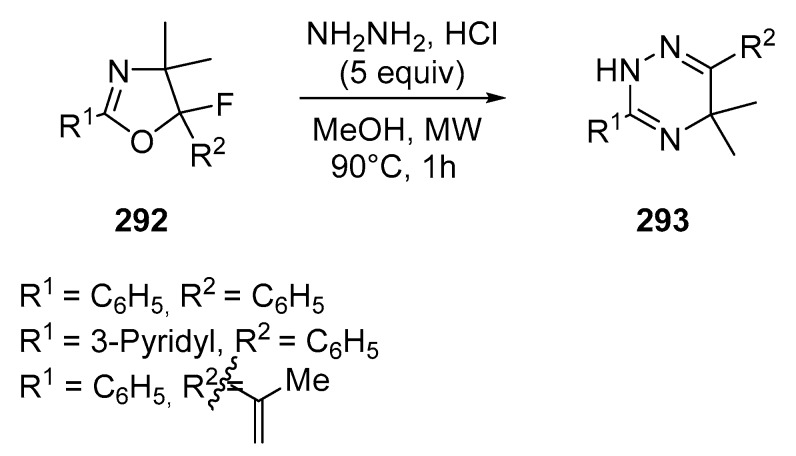
MW-synthesis of 1,2,4-triazines by reaction of oxazolines with hydrazine hydrochloride.

Using another process, Bigot *et al.* [274] have prepared new 1,2,4-triazine derivatives **293** in excellent yields (62%–92%) by the reaction of oxazolines **292** with excess of hydrazine hydrochloride in methanol under MW-irradiation for 1 h at 90 °C (Scheme 67).

On account of the simultaneous presence of four nitrogen atoms in the ring, tetrazines are even more reactive towards nucleophilic agents than triazines, including inverse electron demand in Diels–Alder reactions. Therefore, no data on Diels–Alder reactions of 1,2,3,4-tetrazines and 1,2,3,5-tetrazines have been published, and the term tetrazines will henceforth refer to 1,2,4,5-tetrazines (*s*-tetrazines).

**Scheme 68:**
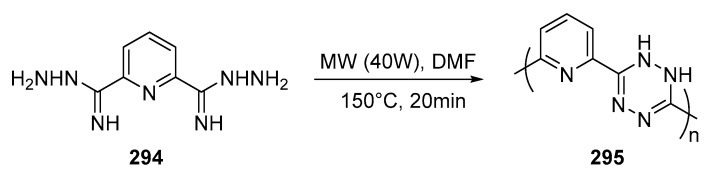
Synthesis of poly(2,6-pyridinediyl-dihydro-*s*-tetrazinylene) under MW-irradiation.

Sagot *et al.* [275] have reported the efficient synthesis of poly(2,6-pyridinediyl-dihydro-*s*-tetrazinylene) (**295**) from pyridine-2,6-diamidrazone (**294**) under MW-activation. The reaction conditions were optimized, and the best result was obtained by the application of an irradiation power of 40 W at 150 °C for 20 min to give the polymer in a 47% yield (Scheme 68).

**Scheme 69:**
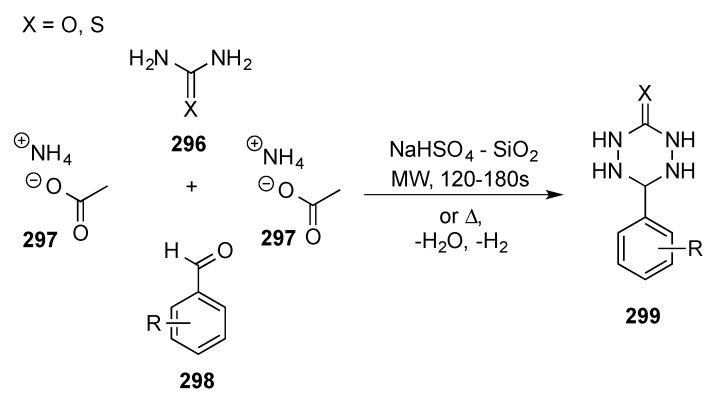
One-pot solvent-free synthesis of tetrazines.

Kanagarajan *et al.* [276] have reported an efficient one-pot solvent-free condensation of urea or thiourea (**296**), diverse aromatic aldehydes **298**, and ammonium acetate (**297**) in the presence of repeatedly usable heterogeneous catalyst NaHSO_4_–SiO_2_ under MW-irradiation. The reactions proceeded faster (120–180 s at 75–78 °C) and with better yields (68%–80%) of 6-aryl-1,2,4,5-tetrazinane-3-thiones(ones) **299** than under conventional heating at 75 °C for 30–70 min (Scheme 69).

## 3. Conclusions

The examples described in this review illustrate that MW-assisted synthesis lead to various heterocyclic systems on an easy and rapid way. We reviewed herein the synthesis of six-membered rings (one to four heteroatoms) and their fused analogues that feature interesting pharmaceutical activities. Many of the early reactions were performed in a dedicated monomode MW-reactor and in simple or “adapted” domestic instruments and some of them also respect the basic rules of green chemistry.

**Figure 18:**
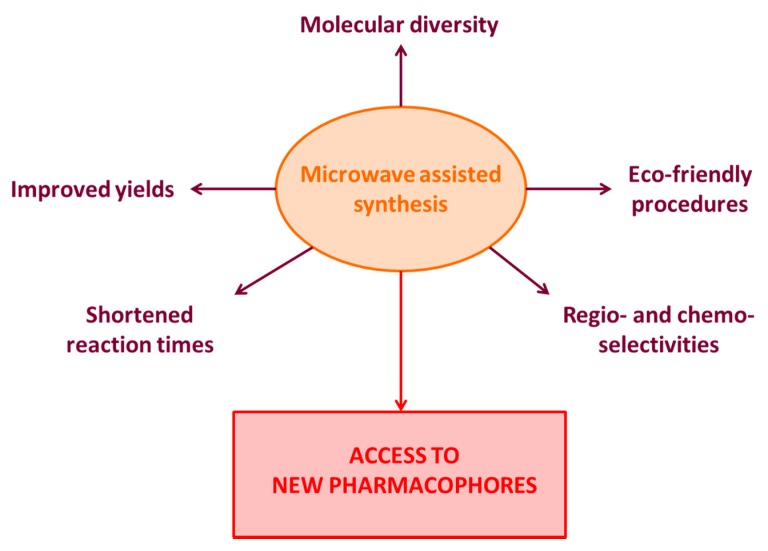
MW-assisted green and efficient processes for the development of new bioactives molecules.

This review is not intended to be exhaustive in its content, but rather to emphasize significant examples where MW irradiation has been either facilitating the synthesis or has afforded a clear methodological advantage over classical and conventional thermal methods. The description of the combination of heterocyclic chemistry and MW irradiation has also shown that performing MW-assisted reactions should be considered with particular attention. A few of these considerations can be applied generally for conducting MW-assisted reactions and include the following: (a) the ratio between the quantity of catalyst or the material and the support or the solvent is very important; (b) for solid starting materials, the use of solid supports can offer operational, economic and environmental benefits over conventional methods. Other aspects can comprise unanswered questions relating to the existence of “intrinsic MW effects,” and the scalability, and overall energy efficiency of this technique (Figure 18).

This technology is still under-used and has the potential to have a large impact on the fields of catalysis, screening, combinatorial chemistry, medicinal chemistry and drug development.

## Abbreviations

The following abbreviations are used in this manuscript:

ATP	Adenosine TriPhosphate
ATR	Ataxia Telangiectasia and Rad3-related protein
BINAP	2,2′-Bis(diphenylphosphino)-1,1′-binaphthyl
DBU	1,8-Diazabicyclo[5.4.0]undec-7-ene
DCB	1,4-Dichlorobenzene
DIPA	Diisopropylamine
DMS	DiMethyl Sulfate
GABA	Gamma-AminoButyric Acid
HATU	1-[Bis(dimethylamino)methylene]-1*H*-1,2,3-triazolo[4,5-*b*]pyridinium-3-oxid hexafluorophosphate
HBTU	2-(1*H*-benzotriazol-1-yl)-1,1,3,3-tetramethyluronium hexafluorophosphate
HOBt	Hydroxybenzotriazole
HPLC	High-Performance Liquid Cchromatography
IC_50_	Half maximal inhibitory concentration
LUMOdiene	Lowest Unoccupied Molecular Orbital in the dienophile
MIC	Minimum Inhibitory Concentration
MW	Microwave
NAD	Nicotinamide Adenine Dinucleotide
NADPH	Nicotinamide Adenine Dinucleotide Phosphate Hydrogen
Nos	The nosyl group
Pbf	2,2,4,6,7-Pentamethyldihydrobenzofuran-5-sulfonyl
PEG	Polyethylene glycol
PPE	PolyPhosphoric Ester
SES	The 2-(trimethylsilyl)ethanesulfonyl group
SNAr	Nucleophilic aromatic substitution
TBAB	Tetrabutylammonium bromide
TBDMS	Tert-butyldimethylsilyl
TEA	Triethylamine
TFA	Trifluoroacetic acid
U-4CR	Ugi four-Component Reaction
UHP	Urea Hydrogen Peroxide
